# Sensitization of the Angiotensin II AT1 Receptor Contributes to RKIP-Induced Symptoms of Heart Failure

**DOI:** 10.3389/fmed.2018.00359

**Published:** 2019-01-09

**Authors:** Stefan Wolf, Joshua Abd Alla, Ursula Quitterer

**Affiliations:** ^1^Molecular Pharmacology, Department of Chemistry and Applied Biosciences, ETH Zurich, Zurich, Switzerland; ^2^Institute of Pharmacology and Toxicology, Department of Medicine, University of Zurich, Zurich, Switzerland

**Keywords:** GRK2, ADRBK1, RKIP, PEBP1, AGTR1, angiotensin II, losartan, heart failure

## Abstract

Inhibition of the G-protein-coupled receptor kinase 2 (GRK2) is an emerging treatment approach for heart failure. Therefore, cardio-protective mechanisms induced by GRK2 inhibition are under investigation. We compared two different GRK2 inhibitors, i.e., (i) the dual-specific GRK2 and raf kinase inhibitor protein, RKIP, and (ii) the dominant-negative GRK2-K220R mutant. We found that RKIP induced a strong sensitization of Gq/11-dependent, heart failure-promoting angiotensin II AT1 receptor signaling. The AT1-sensitizing function of RKIP was mediated by the RKIP-GRK2 interaction because the RKIP-S153V mutant, which does not interact with GRK2, had no effect on AT1-stimulated signaling. In contrast, GRK2-K220R significantly inhibited the AT1-stimulated signal. The *in vivo* relevance of these major differences between two different approaches of GRK2 inhibition was analyzed by generation of transgenic mice with myocardium-specific expression of RKIP and GRK2-K220R. Our results showed that a moderately increased cardiac protein level of RKIP was sufficient to induce major symptoms of heart failure in aged, 8-months-old RKIP-transgenic mice in two different genetic backgrounds. In contrast, GRK2-K220R protected against chronic pressure overload-induced cardiac dysfunction. The AT1 receptor contributed to RKIP-induced heart failure because treatment with the AT1 receptor antagonist, losartan, retarded symptoms of heart failure in RKIP-transgenic mice. Thus, sensitization of the heart failure-promoting AT1 receptor by the RKIP-GRK2 interaction contributes to heart failure whereas dominant-negative GRK2-K220R is cardioprotective. Because RKIP is up-regulated on cardiac biopsy specimens of heart failure patients, the deduced heart failure-promoting mechanism of RKIP could also be relevant for the human disease.

## Introduction

The family of G-protein-coupled receptor kinases (GRKs) initiates the process of signal desensitization by phosphorylation of G-protein-coupled receptors (1, 2). Among different GRKs, the G-protein-coupled receptor kinase 2 (GRK2) is the most important member of the GRK family because functions of GRK2 are indispensable, and complete loss of GRK2 in GRK2-knockout mice is lethal (3). On the other hand, there are multiple lines of evidence, which show that inhibition of exaggerated GRK2 activity in experimental models of heart failure is cardio-protective (4–8). Based on these data, inhibition of GRK2 appears as a promising treatment approach of heart failure (9, 10).

Experimental evidence indicates that a major cardio-protective mechanism induced by GRK2 inhibition relies on re-sensitization of desensitized beta-adrenoceptors in heart failure (9, 10). In addition, several other cardio-protective mechanisms of GRK2 inhibition were deduced, which include improvement of mitochondrial function and promotion of cardiomyocyte survival (11, 12). Prevention of cardiomyocyte death by GRK2 inhibition was partially attributed to enhancement of the pro-survival Raf-Erk pathway (8, 12).

Apart from inhibition of kinase-dependent functions of GRK2, recent data show that kinase-independent functions of GRK2 are also important for cardio-protection. In this respect, the focus lies on the amino-terminal domain of GRK2, which contains a regulator of G-protein signaling (RGS) domain (13, 14). The RGS domain of GRK2 is functional and inhibits Gq/11-mediated signaling (13, 14), which is an established causative factor of myocardial hypertrophy (15). Consequently, by inhibition of pro-hypertrophic Gq/11-mediated signaling (15), the RGS domain of GRK2 has documented cardioprotective activity (16). It is noteworthy that most approaches of GRK2 inhibition leave this cardioprotective RGS domain of GRK2 intact, i.e., the betaARKct, which inhibits GRK2-mediated receptor phosphorylation by scavenging of Gβγ subunits (4, 5), or ATP-site directed kinase inhibitors such as paroxetine (7, 17). Also, the kinase-inactive GRK2-K220R mutant, which acts as a dominant negative GRK2 mutant, has a preserved amino-terminal RGS domain (18), and shows effective RGS domain-mediated inhibition of Gq/11-stimulated signaling (12). But other approaches of GRK2 inhibition target specifically the amino-terminal domain of GRK2 such as the raf kinase inhibitor protein, RKIP, which is the alias for phosphatidylethanolamine-binding protein 1, *PEBP1* (19). Consequently, RKIP could also inhibit the cardioprotective RGS domain of GRK2.

RKIP is an inhibitor of GRK2, which switches from Raf1 to GRK2 by PKC-mediated phosphorylation on serine 153 (19). The serine-153-phosphorylated RKIP interacts with the amino-terminal domain of GRK2 and thereby blunts GRK2-mediated phosphorylation of receptor substrates such as the beta-adrenoceptor (19). On the other hand, by interaction with the RGS domain-containing amino-terminus of GRK2, serine-153-phosphorylated RKIP could also interfere with the cardioprotective Gq/11-inhibitory function of the RGS domain (19). As a consequence, the RKIP-GRK2 interaction, would sensitize signaling stimulated by major Gq/11-coupled, heart failure-promoting GPCRs such as the angiotensin II AT1 receptor (20) by a dual mechanism, which involves GRK2 and RGS domain inhibition.

Because RKIP is up-regulated on cardiac biopsy specimens of failing human hearts (21), these potentially detrimental functions of RKIP are of pathophysiological relevance. We addressed this issue and found that RKIP strongly enhanced signaling stimulated by the Gq/11-coupled AT1 receptor in cells. *In vivo*, a moderately increased cardiac RKIP level induced by transgenic RKIP expression under control of the myocardium-specific alpha-MHC promoter was a sufficient cause for development of major symptoms of heart failure. For comparison, targeting of GRK2 by the dominant-negative GRK2-K220R showed RGS-domain-mediated inhibition of AT1-stimulated signaling in cells, and cardioprotective activity against chronic pressure-overload-induced cardiac dysfunction *in vivo*. In agreement with a causative role of AT1 receptor sensitization in the cardiac phenotype triggered by RKIP, inhibition of the AT1 receptor by the AT1-specific antagonist, losartan, retarded signs of heart failure in RKIP-transgenic mice. Together our data strongly suggest that sensitization of AT1 receptor signaling contributes to RKIP-induced cardiac dysfunction.

## Materials and Methods

### Generation of Transgenic Mice

Different transgenic mouse lines were generated and/or characterized in frame of this study. For transgenic expression of RKIP, the cDNA encoding *PEBP1* (phosphatidylethanolamine-binding protein 1, RKIP) was placed under control of the alpha-myosin heavy chain (alpha-MHC) promoter (8). The cDNA encoding GRK2-K220R (*ADRBK1K220R*) was also inserted into the alpha-MHC plasmid. Plasmid backbone was removed by *Not1* digestion. After pronuclear injection of transgenic DNA (2 ng/μL) into fertilized oocytes isolated from super-ovulated B6 (C57BL/6J) or FVB (FVB/NJ) mice, embryo transfer of 2-cell embryos was performed into 0.5-day pseudo-pregnant CD-1 foster mice. PCR genotyping of offspring was performed at an age of 3 to 4 weeks with ear-punch biopsies. Founder mice of the FO generation with stable integration of the transgenic DNA into the genomic mouse DNA were used for further breeding. Different RKIP-transgenic mouse lines were generated in the B6 (C57BL/6J) background (C57BL/6Tg(MHCPEBP1)1Sjaa; JAX strain ID 911818), and FVB (FVB/NJ) background (FVB/NTg(MHCPEBP1)1Sjaa, JAX strain ID 911819). Tg-GRK2K220R mice were generated in the B6 background (C57BL/6Tg(MHCADRBK1K220R)1Sjaa; JAX strain ID 911825). As indicated, phenotyping of male transgenic mice was performed at an age of 8 months. In addition, 8-week-old male B6 mice were subjected to 2 months of chronic pressure overload imposed by abdominal aortic constriction, AAC (22). At the end of the observation period, AAC-induced chronic pressure overload was controlled and confirmed by an increased systolic aortic pressure (>150 mmHg) as determined by invasive hemodynamic measurement (Micro-Tip^®^ Catheter Pressure Transducer 1F, Millar Instruments). The left ventricular ejection fraction was determined under tribromoethanol anesthesia (250 mg/kg body weight; i.p.; freshly prepared and protected from light) by M-mode echocardiography in the parasternal long-axis view with a Vivid 7 echocardiography equipment and a 12 MHz linear array ultrasound transducer (GE Healthcare). Data were evaluated offline with the EchoPac Pc 3.0 software (GE Healthcare) using the formula of Teichholz to calculate the left ventricular ejection fraction (22). Systolic blood pressure was measured with a PowerLab data acquisition system coupled to a pulse transducer/cuff (AD Instruments). All animal experiments were conducted in agreement with the NIH guidelines, and reviewed and approved by the local committee on animal care and use (Cantonal Veterinary office, Zurich).

### Whole Genome Microarray Gene Expression Profiling

For whole genome microarray gene expression profiling, anesthetized mice (ketamine/xylazine, 100 mg/10 mg/kg) were perfused with PBS, hearts from transgenic mice (Tg-RKIP, Tg-GRK2K220R), and non-transgenic B6 controls were isolated, pulverized under liquid nitrogen, and total RNA was isolated by the RNeasy Midi kit according to the protocol of the manufacturer (Qiagen). RNA purity was confirmed by an absorbance ratio A260/280 of ~2.0. The absence of RNA degradation and RNA quality were further controlled by the presence of bright bands of 18S and 28S ribosomal RNA in denaturing RNA electrophoresis. The RNA was reverse transcribed and processed for whole genome microarray gene expression profiling following the Affymetrix protocol (GeneChip Expression Analysis Technical Manual, rev. 5, Affymetrix Inc., Santa Clara, CA, USA). Fragmented and biotin-labeled cRNA (15 μg/gene chip) in 200 μl of hybridization solution was hybridized to the microarray gene chip (Mouse Genome MG430 2.0 Array, Affymetrix) in a hybridization oven 640 (Affymetrix) for 16 h at 45°C. After washing and staining of gene chips with a Fluidics Station 450 (Affymetrix) according to the GeneChip Expression Analysis Technical Manual, microarrays were scanned with the GeneChip Scanner 7G (Affymetrix), and signals were processed with a target value of 300 using GCOS software version 1.4 (Affymetrix). Cardiac RNA from three mice was pooled for one gene chip, and two gene chips are presented for each group. Such an approach is feasible with inbred mouse lines due to negligible intra-individual variability (23). Probe sets with significantly different signal intensities were identified by TIGR MeV (*p* < 0.01, just alpha, ≥2-fold difference between probe sets with call present and/or signal intensity ≥100). These selection criteria were specifically validated for treatment effects (23) and follow the guidelines of the MicroArray Quality Control (MAQC) project for the identification of reproducible gene lists (24, 25). Results were similarly obtained with GCOS/RMA-processed data using GeneSpring GX software (Agilent Technologies Inc., Santa Clara, CA, USA). Selected transcripts were analyzed after reverse transcription by quantitative real time qRT-PCR using a LightCycler 480 Instrument (Roche). Primers used for qRT-PCR of PEBP1 expression in Tg-RKIP mice did not amplify the mouse *Pebp1* (PEBP1 forward 5′-GCA GCA CCC GCT GCA TGT CAC-3′; PEBP1 reverse 5′-CTC GTC ACA CTT TAG CGG CCT G-3′). Microarray gene expression data were deposited to the NCBI GEO database (accession number GSE 120020).

### Immunoblot Detection and Immunohistology

Cardiac protein levels of RKIP (PEBP1, Pebp1), GRK2, and Pparg were determined by immunoblot analysis of hearts isolated from Tg-RKIP and Tg-GRK2K220R mice. The respective non-transgenic mice (B6 or FVB) were used as controls. Anesthetized mice (ketamine/xylazine, 100 mg/10 mg/kg) were perfused with PBS, hearts were isolated and immediately frozen in liquid nitrogen. For protein detection in immunoblot, hearts were pulverized under liquid nitrogen, and proteins were extracted with RIPA buffer supplemented with protease/phosphatase inhibitors. Pellets from AT1 receptor-expressing HEK cells with transfection of expressing plasmids encoding RKIP and GRK2 or mock-transfected cells as indicated were similarly extracted. Particulate material was removed by centrifugation followed by protein precipitation/delipidation with acetone/methanol (12:2; final concentration 83%) for 90 min at 4°C. The precipitate was collected by centrifugation and washed 3 times with 0.2 ml of ice-cold acetone. The pellet was dissolved in SDS sample buffer containing 2% SDS, 0.1 M DTT and 6 M urea by incubation for 90 min at room temperature. Samples were stored at −70°C for further use. Immunoblot detection of proteins was performed after separation of proteins by SDS-PAGE and electrophoretic protein transfer to PVDF membranes. For immunoblot detection of proteins, we used affinity-purified antibodies or F(ab)_2_ fragments of the respective antibodies pre-absorbed on mouse/human serum proteins. Bound antibody was visualized with F(ab)_2_ fragments of peroxidase-coupled secondary antibodies (pre-absorbed on mouse/human serum proteins) or peroxidase-coupled protein A followed by chemiluminescent western blot detection (ECL Plus or ECL Prime; Amersham). Histological analyses were performed with paraffin-embedded cardiac sections of the different transgenic mouse lines and compared to non-transgenic controls (8). Myocyte cross-sectional diameter was determined by histomorphometrical analysis of hematoxylin-eosin-stained longitudinal sections of 5 μm thickness. The mean cardiomyocyte cross-sectional diameter (CSD) in the left ventricular free wall was determined by computerized image analysis (Image J) by an observer, who was blinded to the mouse genotype. Cardiomyocyte diameter quantification used 6 different hearts/group with five separate fields of cells on each heart. A total of 100 cardiomyocytes with centered nuclei were evaluated per heart. Immunohistological detection of RKIP was performed on longitudinal cardiac sections after antigen retrieval with affinity-purified polyclonal antibodies raised against full-length, recombinant RKIP. Antibody incubation was performed for 60 min at 37°C in blocking buffer (PBS, pH 7.4, supplemented with 5% bovine serum albumin, 0.05% Tween-20), and unbound antibodies were removed by three washing steps with PBS supplemented with 0.05% Tween 20. After incubation with peroxidase-conjugated secondary antibody (dilution 1:500, Dianova, Hamburg) and removal of unbound antibodies by washing steps, bound antibody was visualized by an enzyme substrate reaction (DAB Enhanced Liquid Substrate System, Sigma). Myocardial necrosis was determined by von Kossa staining (Calcium stain kit, modified Von Kossa No. KT028, Diagnostic Biosystems Pleasanton, CA, USA). A Leica DMI6000 microscope equipped with a DFC 420 camera was used for imaging of (immuno)-histological sections.

### Antibodies

The study used the following antibodies for immunoblotting and immunohistology: polyclonal anti-RKIP antibodies were raised in rabbit against full-length, recombinant RKIP (8); polyclonal anti-phospho-S153-RKIP antibodies were raised in rabbit against an epitope in RKIP encompassing phospho-serine-153 (sc-32623; Santa Cruz Biotechnoloy Inc.); polyclonal anti-GRK2 antibodies were raised in rabbit against full-length, recombinant GRK2 (ADRBK1) protein expressed in and purified from Sf9 cells (12); polyclonal anti-Gnb/GNB antibodies were raised in rabbit against purified GNB (8); polyclonal anti-Pparg antibodies were raised in rabbit against a peptide encompassing amino acids 8-106 of PPARG (Santa Cruz Biotechnology Inc.); polyclonal antibodies against phospho-Ser-273 PPARG were raised against a synthetic phosphopeptide encompassing the phosphorylation site of serine-273 of PPARG (bs-4888R; BIOSS antibodies).

### Biochemical Assays

For lipid analysis, frozen hearts were pulverized under liquid nitrogen, and cardiac lipids were extracted (26). Gas chromatography (GC) analysis of cardiac lipids was performed on a gas chromatograph (Focus, Thermo Scientific) equipped with a DB-23 column (Agilent J&W). After transesterification of cardiac lipids, fatty acid methyl esters (FAMEs) were detected by a flame ionization detector and identified by comparison with a mixture of commercial FAME reference standards (Supelco 37 component FAME mix, Sigma Aldrich). For quantitative lipid analysis, an internal standard was included. Cardiac TAG (triacylglycerol) contents were determined by a commercial kit (TR0100; Sigma). Cardiac DAG (diacylglycerol) and ceramide contents were quantified by the DAG kinase method (8, 27). The urinary albumin to creatinine ratio (ACR) was determined by commercial kits (BCG albumin assay kit MAK124; creatinine assay kit MAK080; Sigma). Number of cardiac AT1 receptor binding sites was determined with cardiac membranes by radioligand binding assay with Sar^1^,[^125^I]Tyr^4^,Ile^8^-angiotensin II (2200 Ci/mmol) in the presence and absence of a 1,000-fold molar excess of losartan to determine non-specific binding. HEK cells were cultured and transfected with expression plasmids encoding the AT1 receptor (*AGTR1*), GRK2 (*ADRBK1*), GRK2-K220R, RKIP (*PEBP1*), and RKIP-S153V as described (12, 28). Total cellular inositol phosphate levels were determined of HEK cells with stable AT1 receptor expression, which were transfected with the indicated expression plasmids (28). Endogenously expressed human RKIP (*PEBP1*) of AT1 receptor-expressing HEK cells, and transgenic RKIP (*PEBP1*) of Tg-RKIP mice were down-regulated by transfection/transduction of a plasmid/lentivirus with an engineered pre-miRNA, which targets *PEBP1* by RNA interference (RNAi). The following double-stranded oligonucleotides, which encode an engineered pre-miRNA targeting human RKIP (*PEBP1*) by RNAi were inserted into pcDNA6.2-GW/miR for down-regulation of endogenous RKIP in HEK cells and pLenti6/V5-Dest Gateway (Invitrogen) for generation of a lentiviral expression plasmid to down-regulate human RKIP in Tg-RKIP mice: miPEBP1-top 5′-TGC TGT GTA GAG CTT CCC TGA ATC AAG TTT TGG CCA CTG ACT GAC TTG ATT CAG AAG CTC TAC A−3′; and miPEBP1-bottom 5′-CCT GTG TAG AGC TTC TGA ATC AAG TCA GTC AGT GGC CAA AAC TTG ATT CAG GGA AGC TCT ACA C−3′. For lentiviral transduction of mice, a pseudotyped lentivirus was generated as described (8). By a similar RNAi-mediated approach, endogenously expressed GRK2 of HEK cells was down-regulated (12). Neonatal mouse cardiomyocytes were isolated from Tg-RKIP mice, Tg-GRK2-K220R mice, and non-transgenic B6 controls as described (8). Cellular cAMP levels of isolated neonatal cardiomyocytes (without and with stimulation by 100 nM isoproterenol) were determined using a commercial kit (CA200, Sigma Aldrich).

### Statistical Analysis

Data are presented as mean ± s.d. Statistical significance between two groups was calculated by the unpaired, two-tailed Student's *t*-test. For comparisons between more than two groups, analysis of variance followed by a Post-test as indicated was performed. Statistical significance was set at a *p*-value of <0.05 unless otherwise stated. Statistical evaluation was performed with GraphPad PRISM 7.0a. Whole genome gene expression data were analyzed by TIGR Multi Experiment Viewer MeV.

## Results

### RKIP Promotes AT1 Receptor Sensitization Whereas GRK2-K220R Inhibits AT1-Stimulated Signaling

**Figure 1:**
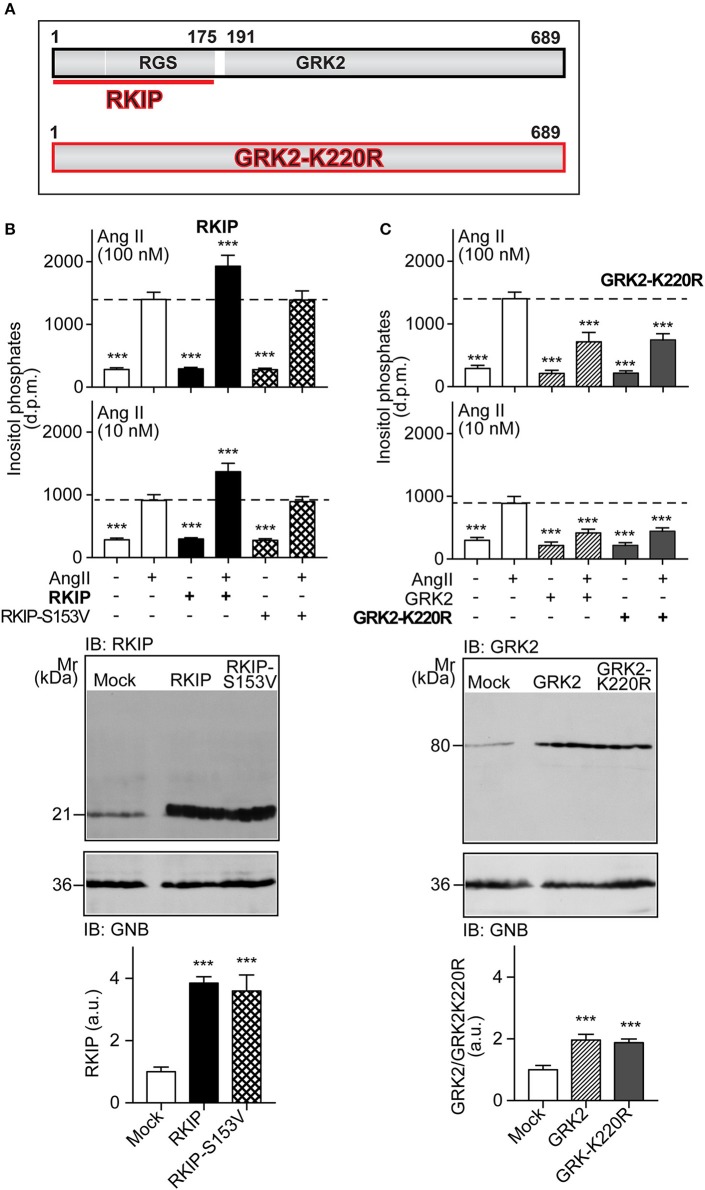
RKIP promotes AT1 receptor sensitization whereas GRK2-K220R inhibits AT1-stimulated signaling. **(A)** Scheme of GRK2 and the amino-terminal RKIP interaction site (upper panel) compared to the dominant-negative GRK2-K220R mutant (lower panel). **(B,C)** Total inositol phosphate levels of AT1 receptor-expressing HEK cells stimulated without (–) or with (+) angiotensin II (100 and 10 nM) and transfected without (–) or with (+) RKIP and RKIP-S153V **(B)**, or GRK2 and GRK2-K220R **(C)** as indicated (upper panels). Data are expressed as mean ±s.d. (*n* = 8; ^***^p < 0.001 vs. column 2; Tukey's test).The lower panels show immunoblots, which detect RKIP **(B)** and GRK2 **(C)** proteins of HEK cells expressing the indicated proteins Immunoblot detection of GNB was used as a loading control (±s.d.; *n* = 4; ^***^p < 0.001 vs. mock; Tukey's test). See also Supplementary Figures 1A,B.

RKIP interacts with the amino-terminal domain of GRK2 and thereby blunts the interaction of GRK2 with receptor substrates, with subsequent inhibition of receptor phosphorylation and desensitization [Figure 1A and (19)]. The amino-terminal domain of GRK2 also contains an intact RGS domain (Figure 1A), which blunts Gq/11-dependent signal transduction (13, 14). We investigated the impact of RKIP on signaling mediated by the heart failure-promoting, Gq/11-coupled angiotensin II AT1 receptor. For *in vitro* experiments, we used HEK cells as a model system because these cells are widely used to study mechanisms of signal transduction, notably signaling events triggered by the AT1 receptor, GRK2 and RKIP (12, 19, 28). We found that RKIP led to a significantly enhanced angiotensin II AT1-stimulated inositol phosphate generation in HEK cells (Figure 1B). This signal sensitization of the AT1 receptor was mediated by the RKIP-GRK2 interaction because the RKIP-S153V mutant, which cannot switch from Raf1 to GRK2 (19), had no effect on the AT1 signal (Figure 1B). As a control, RKIP and RKIP-S153V protein levels were comparable (Figure 1B).

In contrast to RKIP, the GRK2-K220R mutant, which acts as a dominant-negative mutant of GRK2 (Figure 1A), led to a strong inhibition of the AT1-stimulated signal (Figure 1C). Inhibition of the Gq/11-coupled AT1 receptor was similarly observed with wild-type GRK2 (Figure 1C). This experiment confirms that the Gq/11-inhibitory activity of GRK2 is a kinase-independent effect.

### Physiological RKIP Levels Are Sufficient to Sensitize AT1-Stimulated Signaling in Cells

**Figure 2:**
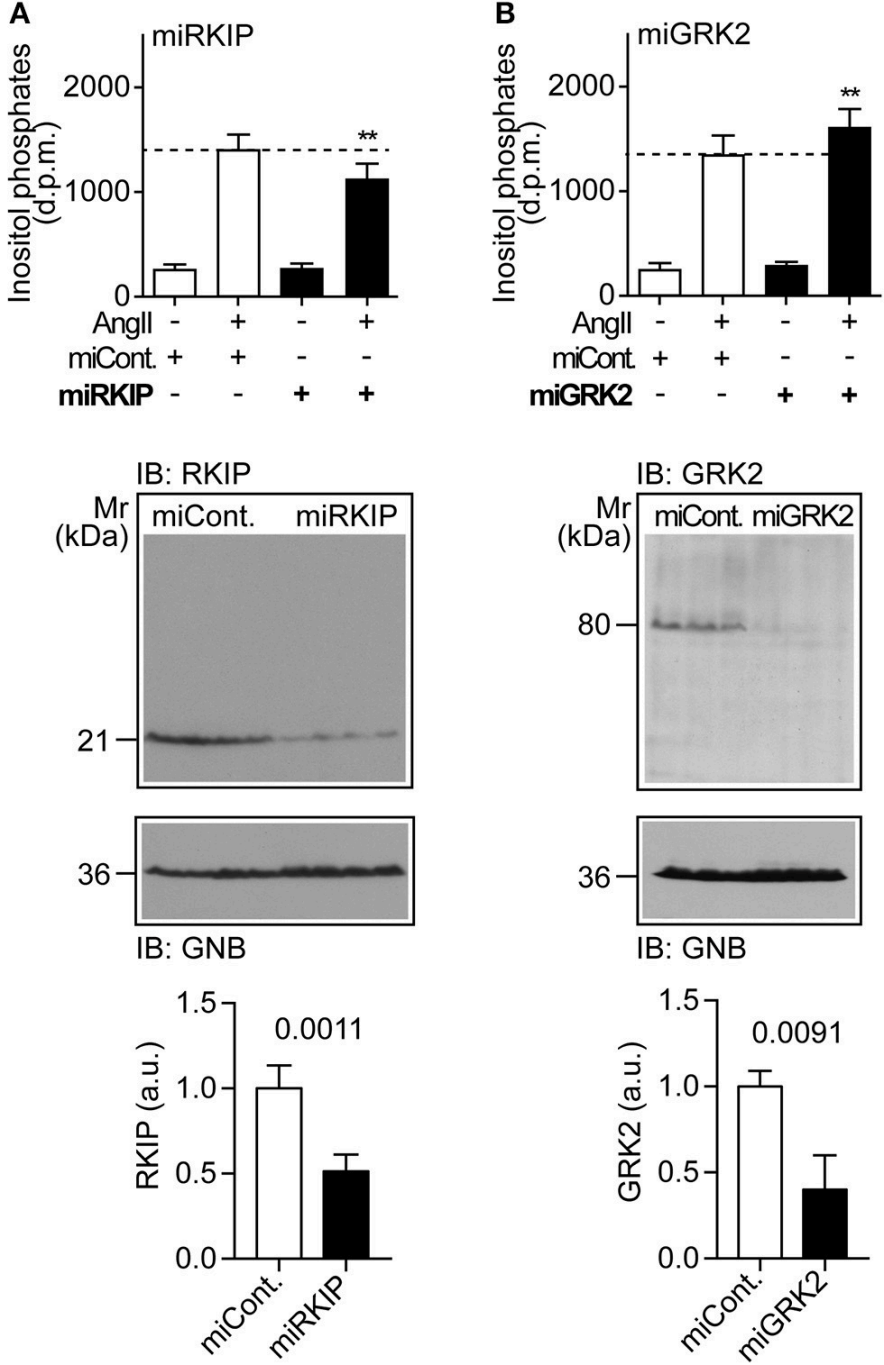
Physiological RKIP levels are sufficient to sensitize AT1-stimulated signaling in cells. **(A,B)** Total inositol phosphate levels of AT1 receptor-expressing HEK cells stimulated without (–) or with (+) angiotensin II (100 nM) and transfected without (–) or with (+) a control pre-miRNA (miCont) or a pre-miRNA targeting endogenously expressed RKIP (miRKIP) or GRK2 (miGRK2) by RNAi as indicated. Data represent mean ± s.d. (*n* = 8; ^**^p < 0.01 vs. column 2; Dunnett's test). The lower panels confirm RNAi-mediated down-regulation of endogenously expressed proteins by immunoblot detection of RKIP **(A)** and GRK2 **(B)**. Data represent mean ± s.d. (*n* = 4). See also Supplementary Figures 2A,B.

We asked whether physiological protein levels of RKIP are sufficient to sensitize the AT1-stimulated response, and down-regulated endogenously expressed RKIP by transfection of a pre-miRNA targeting RKIP (*PEBP1*) by RNAi. Upon RNAi-mediated down-regulation of endogenously expressed RKIP, the AT1-stimulated inositol phosphate generation was significantly decreased (Figure 2A). As a control, down-regulation of endogenously expressed RKIP was confirmed by immunoblot analysis (Figure 2A). This experiment shows that physiological RKIP levels are sufficient to sensitize the angiotensin II AT1 receptor because down-regulation of RKIP led to a decreased AT1 response.

We also down-regulated the endogenously expressed GRK2 by RNAi (Figure 2B). In contrast to RKIP, down-regulation of GRK2 led to a significantly increased AT1-stimulated signal (Figure 2B). This finding shows that endogenously expressed GRK2 inhibits the Gq/11-coupled AT1 receptor. Together these experiments reveal that RKIP sensitizes the heart failure-promoting AT1 receptor whereas endogenously expressed GRK2 and the kinase-deficient GRK2-K220R mutant inhibit AT1-stimulated signaling.

### Myocardium-Specific Expression of RKIP Induces Cardiac Dysfunction Whereas GRK2-K220R Improves Cardiac Function

**Figure 3:**
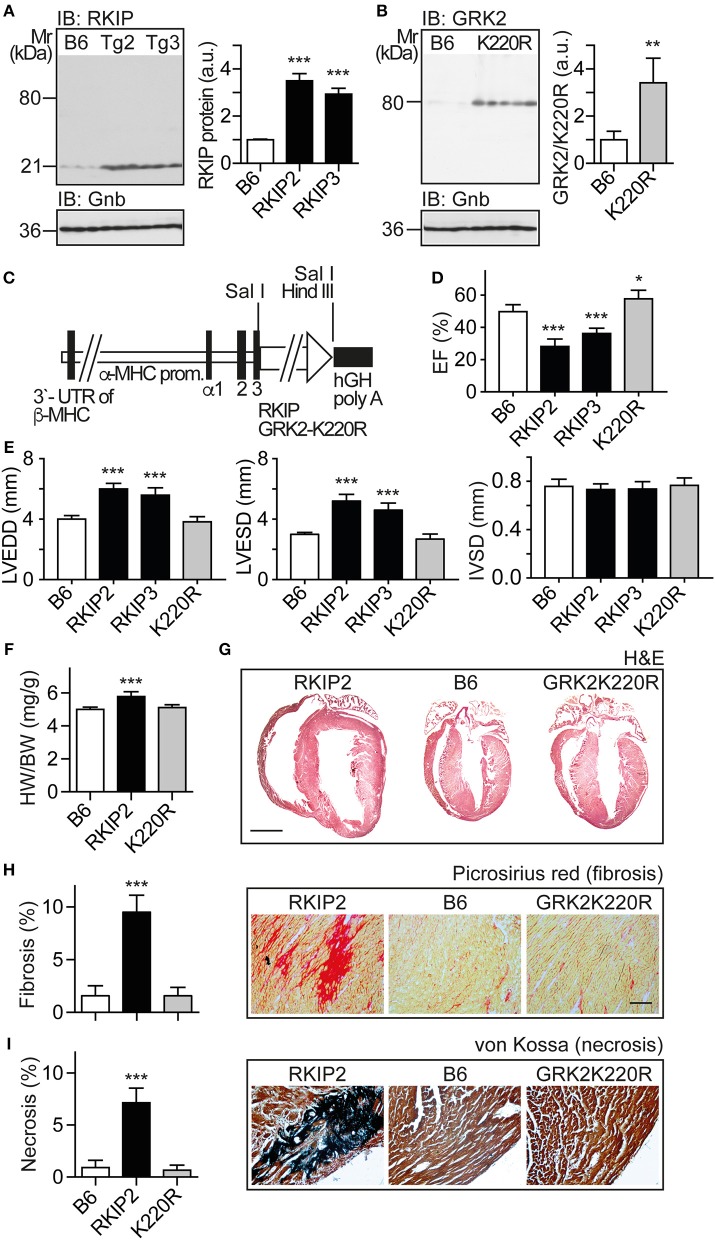
Myocardium-specific expression of RKIP induces cardiac dysfunction whereas GRK2-K220R improves cardiac function. **(A)** Immunoblot detection of RKIP in non-transgenic B6 and two different Tg-RKIP mouse lines, Tg-RKIP2 and Tg-RKIP3 (±s.d.; *n* = 3; ^***^p < 0.001 vs. B6; Dunnett's test). **(B)** Immunoblot detection of GRK2/GRK2-K220R in non-transgenic B6 and Tg-GRK2K220R mice [±s.d., *n* = 3 (B6) and *n* = 5 (K220R); ^**^p = 0.0097]. **(C)** Scheme of expression plasmid used for generation of Tg-RKIP and Tg-GRK2K220R mice with myocardium-specific expression of RKIP and GRK2-K220R under control of the alpha-MHC promoter. **(D)** Left ventricular ejection fraction (EF) of different eight-months-old transgenic mouse lines compared to age-matched, non-transgenic B6 controls (±s.d., *n* = 5; ^***^p < 0.001, and ^*^p < 0.05 vs. B6; Dunnett's test). **(E)** Left ventricular end-diastolic diameter (left), left ventricular end-systolic diameter (middle), and interventricular septal thickness (right) of B6, Tg-RKIP2, Tg-RKIP3, and Tg-GRK2K220R mice (±s.d., *n* = 5; ^***^p < 0.001 vs. B6; Dunnett's test). **(F)** Heart-to-body weight ratio of different transgenic lines compared to non-transgenic B6 controls (±s.d.; *n* = 6; ^***^p < 0.001 vs. B6; Dunnett's test). **(G)** Histological assessment of cardiac sections from a Tg-RKIP2 mouse, a non-transgenic B6 mouse and a Tg-GRK2K220R mouse. Sections were stained with hematoxylin-eosin (H&E) and are representative of six mice/group (bar: 2 mm). (H,I) Cardiac fibrosis was determined by picrosirius red staining **(H)**, and myocardial necrosis was assessed by von Kossa stain **(I)** of Tg-RKIP2 and Tg-GRK2K220R mice compared to non-transgenic B6 mice. Left panels show quantitative data evaluation (±s.d.; *n* = 6 mice/group; ^***^p < 0.001 vs. B6; Dunnett's test), and right panels show representative histological sections (bar: 40 μm). See also Supplementary Figures 3A,B.

In view of these major differences between two different approaches of GRK2 inhibition with RKIP and GRK2-K220R, we compared the two inhibitors *in vivo*. We generated transgenic mice with myocardium-specific expression of RKIP and GRK2-K220R under control of the alpha-MHC promoter in B6 background (Figures 3A–C). The cardiac RKIP protein levels of two different RKIP-transgenic mouse lines, Tg-RKIP2 and Tg-RKIP3, were increased 3.5-fold and 2.9-fold, respectively, over non-transgenic B6 controls (Figure 3A). RKIP-transgenic mice were compared with Tg-GRK2K220R mice, which showed a 3.4 ± 0.46-fold increased cardiac GRK2-K220R protein level compared to non-transgenic B6 controls (Figure 3B).

We analyzed the cardiac phenotype of 8-months-old transgenic mice with myocardium-specific expression of RKIP and GRK2-K220R, respectively (Figure 3C). Eight-months-old Tg-RKIP mice developed signs of heart failure in the absence of additional stressors (Figures 3D–I). Heart failure in Tg-RKIP mice was documented by a significantly decreased left ventricular ejection fraction of 28.2 ± 4.2% and 37.0 ± 3.6% in Tg-RKIP2 and Tg-RKIP3 mouse lines, respectively (Figure 3D). In addition to cardiac dysfunction, echocardiographic data showed that Tg-RKIP hearts were enlarged with significantly increased left ventricular end-diastolic (LVEDD) and end-systolic diameters (LVESD) compared to non-transgenic B6 mice while interventricular septal thickness at diastole (IVSD) was not significantly different (Figure 3E). The phenotype of cardiac hypertrophy in Tg-RKIP mice compared to non-transgenic B6 controls was further documented by an increased heart-to-body weight ratio (Figure 3F). Histological analysis of Tg-RKIP2 hearts confirmed these data and showed a phenotype of cardiac hypertrophy with dilatation (Figure 3G). Histological analysis further documented significant cardiac fibrosis and cardiomyocyte necrosis in Tg-RKIP2 hearts (Figures 3H,I). This histological characterization of Tg-RKIP mice complements functional data and shows that cardiac dysfunction in Tg-RKIP mice was accompanied by severe myocardial damage, which develops during the pathogenesis of heart failure as a consequence of maladaptive cardiac remodeling.

In contrast to Tg-RKIP mice, transgenic mice with myocardium-specific expression of the kinase-inactive GRK2-K220R mutant had a slightly improved cardiac function and showed no signs of cardiac hypertrophy (Figures 3D–F). The histological analysis confirmed the normal phenotype of Tg-GRK2K220R hearts, which was not different from non-transgenic B6 controls (Figures 3G–I). Notably, there was no evidence of cardiac hypertrophy and cardiac dilatation in Tg-GRK2K220R mice (Figures 3E–G).

### Major Symptoms of Heart Failure With Pulmonary Congestion and Renal Dysfunction in Tg-RKIP Mice

**Figure 4:**
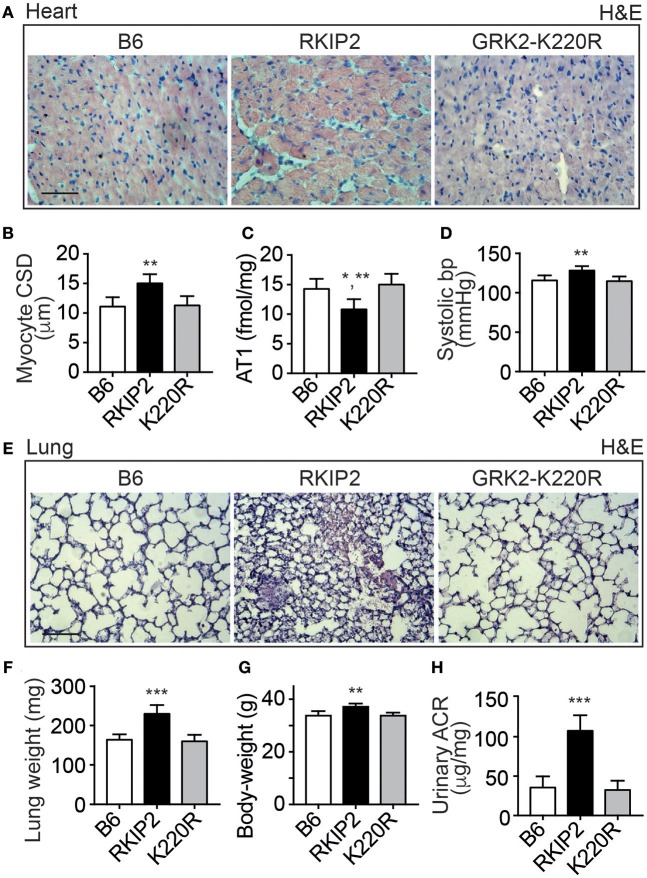
Major symptoms of heart failure with pulmonary congestion and renal dysfunction in Tg-RKIP mice. **(A,B)** Cardiomyocyte cross-sectional diameter was determined with histological cardiac sections of B6, Tg-RKIP2, and Tg-GRK2K220R mice **(A)**. **(B)** Shows quantitative data evaluation. Sections were stained with hematoxylin and eosin (H&E) and are representative of six mice each (bar: 20 μm). **(C,D)** Cardiac AT1 receptor content **(C)**, and systolic blood pressure **(D)** in B6, Tg-RKIP2 and Tg-GRK2K220R mice. **(E)** Histological lung sections show heart failure-related lung remodeling with chronic pulmonary congestion and thickening of alveolar septa in Tg-RKIP2 mice compared to healthy non-transgenic B6 and Tg-GRK2K220R mice. Sections were stained with hematoxylin and eosin (H&E) and are representative of six mice each (bar: 40 μm). **(F)** Increased lung weight of Tg-RKIP2 compared to B6 and Tg-GRK2K220R mice. **(G,H)** Increased body weight **(G)**, and renal insufficiency with proteinuria **(H)** of Tg-RKIP2 mice compared to non-transgenic B6 and Tg-GRK2K220R mice. Data represent the mean ±s.d. [*n* = 6; ^*^p < 0.05 vs. B6; ^**^p < 0.01 vs. B6 **(B,D,G)** and K220R **(B,C,D,G)**; ^***^p < 0.001 vs. B6 and K220R; Tukey's test]. See also Supplementary Figure 4.

Cardiac dysfunction of Tg-RKIP mice was accompanied by major symptoms of heart failure. RKIP-mediated sensitization of pro-hypertrophic AT1-stimulated signaling in Tg-RKIP2 hearts could contribute to the significantly enlarged cardiomyocyte cross-sectional diameter (Figures 4A,B), which is a major factor accounting for maladaptive remodeling in heart failure. In agreement with excessive AT1-stimulated signaling and complementary to human heart biopsy specimens from heart failure patients (29, 30), the AT1 receptor was down-regulated in Tg-RKIP2 hearts (Figure 4C). This decrease in cardiac AT1 receptor content could be a direct consequence of RKIP-induced sensitization of AT1-stimulated signaling (cf. Figure 1), because AT1-stimulated signaling is a causative factor of AT1 down-regulation (31, 32). In contrast, Tg-GRK2K220R mice did not show cardiomyocyte hypertrophy (Figures 4A,B). Moreover, transgenic GRK2-K220R expression did not lead to decreased cardiac AT1 receptor levels (Figure 4C).

Symptoms of heart failure with severe cardiac dysfunction in Tg-RKIP2 mice were accompanied by an increased systolic blood pressure (Figure 4D). Concomitantly, histological lung sections of Tg-RKIP2 mice showed signs of pulmonary congestion with alveolar septal thickening, which was absent in healthy, non-transgenic B6 controls, and Tg-GRK2K220R mice (Figure 4E). Pulmonary congestion was confirmed by an increased lung weight and lung-to-body weight ratio of Tg-RKIP2 mice compared to B6 and Tg-GRK2-K220R mice (Figure 4F, Supplementary Figure 4). Congestive heart failure was further documented by a significantly increased body weight of Tg-RKIP2 mice (Figure 4G). In addition, heart failure in Tg-RKIP2 mice led to symptoms of renal insufficiency with significant proteinuria (Figure 4H). Taken together, a moderately increased cardiac RKIP level is a sufficient cause for the development of major systemic symptoms of chronic congestive heart failure in aged Tg-RKIP mice.

### Up-Regulation of Heart Failure-Related *Pparg* Target Genes in Tg-RKIP Hearts

**Figure 5:**
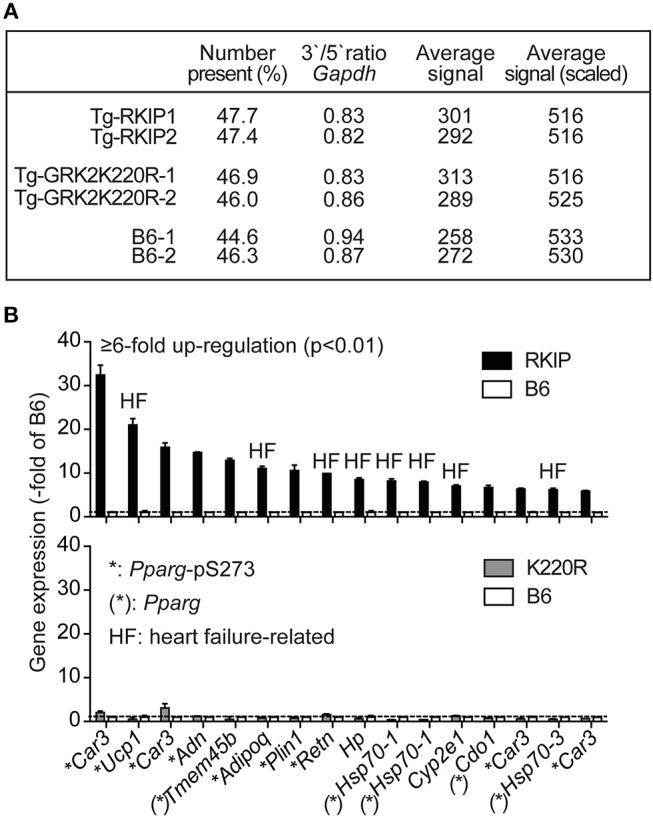
Up-regulation of heart failure-related *Pparg* target genes in Tg-RKIP hearts. **(A,B)** Cardiac whole genome microarray gene expression profiling **(A)** identified highly up-regulated probe sets (≥6-fold up-regulation compared to B6 with *p* < 0.01) in Tg-RKIP hearts (**B**, upper panel). None of these probe sets was significantly regulated in Tg-GRK2K220R hearts (**B**, lower panel). Gene expression data are expressed as -fold of B6 (±s.d., *n* = 2 gene chips/group with total RNA isolated from *n* = 3 hearts/gene chip). Highly up-regulated genes (≥6-fold up-regulation in Tg-RKIP hearts compared to B6 hearts; *p* < 0.01; with call present and/or signal intensity ≥100) in Tg-RKIP hearts have documented relationship to heart failure (HF), are *Pparg* targets [(^*^): *Pparg*)], and/or are induced by *Pparg* serine-273 phosphorylation (^*^:*Pparg*-pS273).

We performed cardiac whole genome microarray gene expression profiling to analyze mechanisms underlying the different phenotype caused by transgenic expression of two GRK2 inhibitors, RKIP and GRK2-K220R (Figure 5A). Microarray gene expression analysis identified highly up-regulated genes (≥6-fold) in Tg-RKIP hearts compared to non-transgenic B6 controls (Figure 5B). Most of these highly up-regulated genes are targets of the adipogenic and heart failure-promoting transcription factor, peroxisome proliferator-activated receptor-gamma, *Pparg*, and have documented relationship to heart failure (Figure 5B). In contrast to Tg-RKIP hearts, transgenic expression of GRK2-K220R did not up-regulate any of these heart failure-promoting genes (Figure 5B).

### Activation of *Pparg*, and Cardiotoxic Lipid Load in Tg-RKIP but Not in Tg-GRK2K220R Hearts

**Figure 6:**
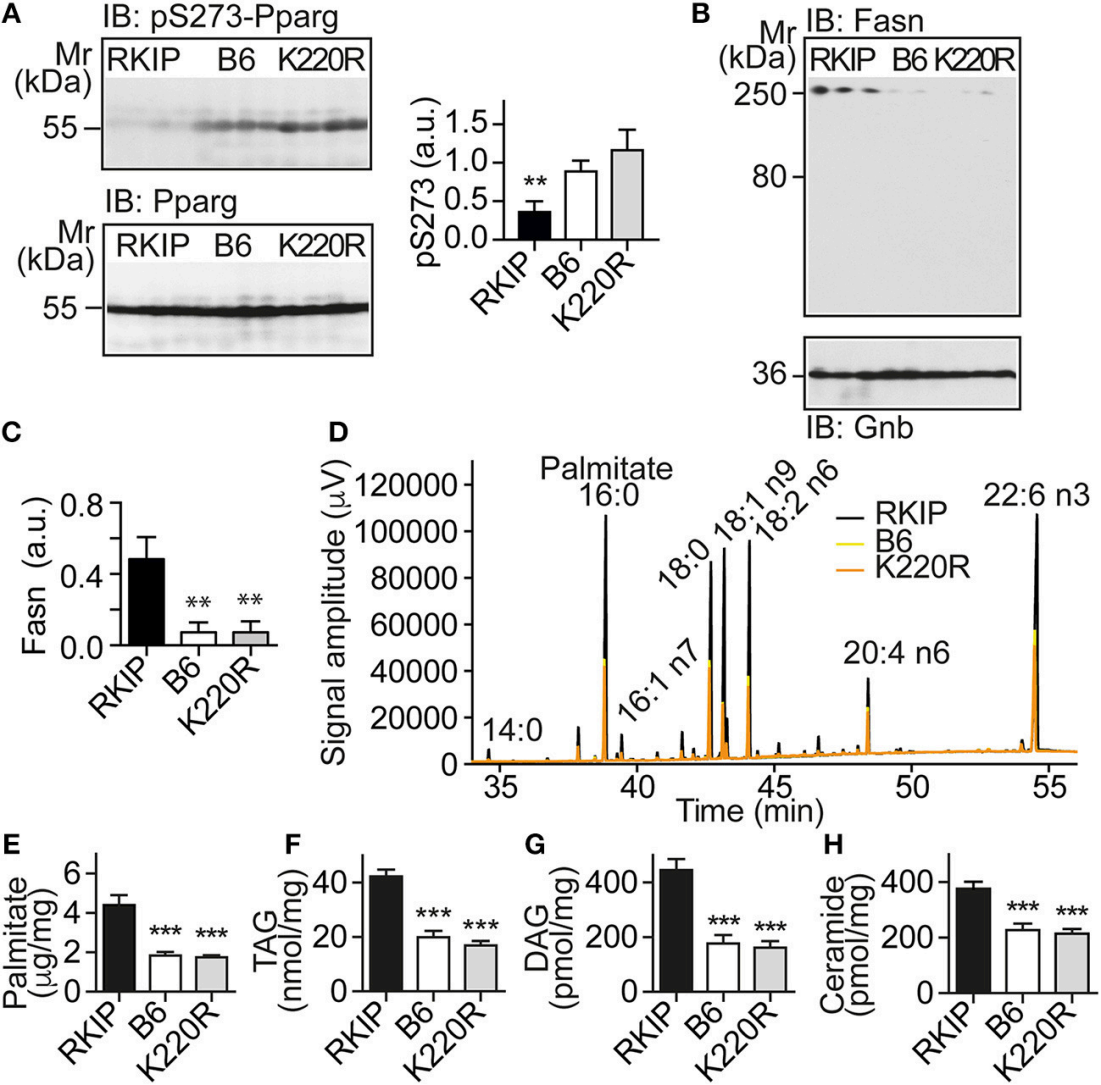
Activation of *Pparg*, and cardiotoxic lipid load in Tg-RKIP but not in Tg-GRK2K220R hearts. **(A)** Immunoblot detection of Pparg phosphorylated on serine-273 (pS273-Pparg) in Tg-RKIP, non-transgenic B6 and Tg-GRK2K220R hearts (±s.d.; *n* = 4 hearts/group; ^**^p < 0.01 vs. B6 and K220R; Tukey's test). The lower panel shows the total cardiac Pparg protein content. **(B,C)** Immunoblot detection of Fasn in Tg-RKIP, non-transgenic B6 and Tg-GRK2K220R hearts (±s.d.; *n* = 3 hearts/group; ^**^p < 0.01 vs. RKIP; Tukey's test). **(D)** GC analysis of cardiac lipids of Tg-RKIP, Tg-GRK2K220R, and non-transgenic B6 hearts. **(E**-**H)** Cardiac lipid analysis detects increased cardiac contents of palmitate **(E)**, TAG **(F)**, DAG **(G)**, and ceramide **(H)** in Tg-RKIP hearts (±s.d.; *n* = 7; ^***^p < 0.001 vs. RKIP; Dunnett's test). See also Supplementary Figures 5A–C.

The up-regulation of *Pparg* targets was accompanied by a significantly decreased phosphorylation of Pparg on serine-273 in Tg-RKIP hearts (Figure 6A). This finding is relevant because dephosphorylation of Pparg on serine-273 leads to an enhanced *Pparg* transcriptional activity (8, 33). In contrast, *Pparg* was not activated in Tg-GRK2K220R hearts, and the content of serine-273-phosphorylated Pparg in Tg-GRK2K220R hearts was comparable to B6 control level (Figure 6A).

Together with an enhanced activation of the adipogenic *Pparg*, Tg-RKIP hearts had an increased cardiac protein content of the major palmitate-synthesizing enzyme and *Pparg* target, fatty acid synthase, Fasn (Figures 6B,C).

Lipid analysis showed that cardiac lipid contents of palmitate, TAG (triacylglycerol), DAG (diacylglycerol), and ceramide were also significantly higher in Tg-RKIP hearts compared to non-transgenic B6 control hearts and Tg-GRK2K220R hearts (Figures 6D–H). In addition to palmitate, DAG and ceramide are also cardiotoxic lipid species, which could contribute to cardiac degeneration and cardiomyocyte loss in Tg-RKIP hearts (8, 27, 34).

### RKIP Induces Symptoms of Heart Failure in FVB Background

**Figure 7:**
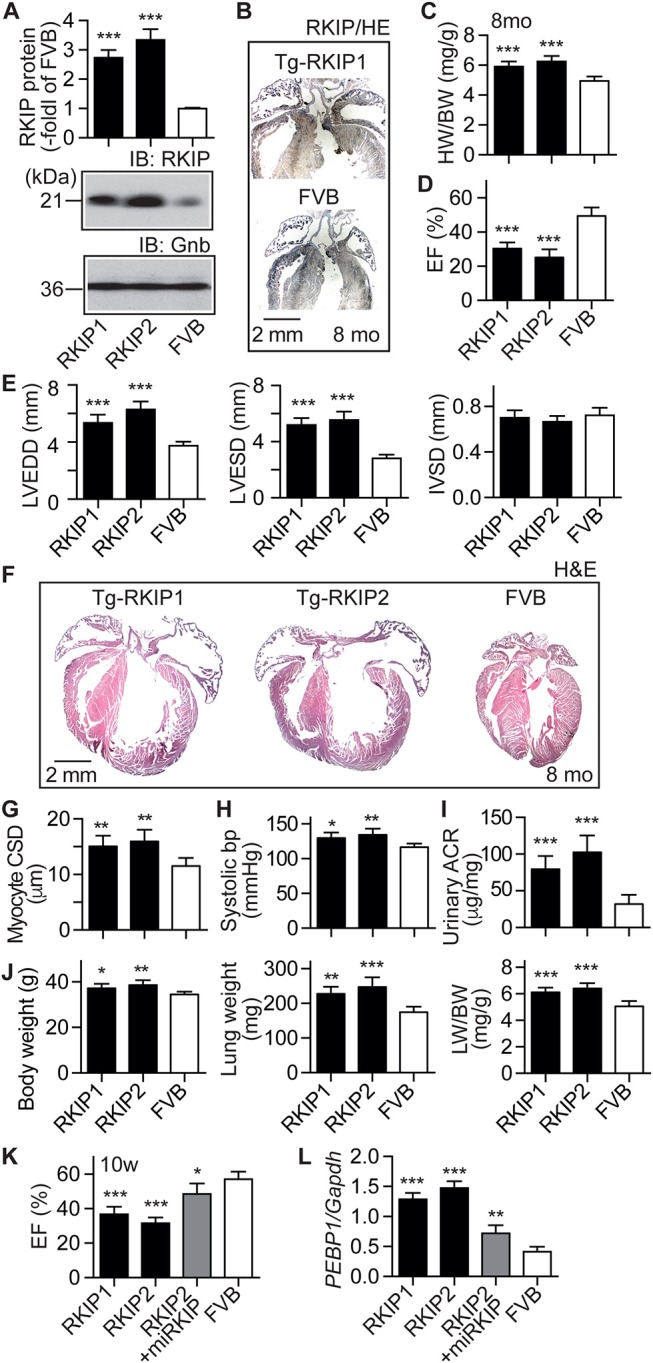
RKIP induces symptoms of heart failure in FVB background. **(A)** Cardiac RKIP protein levels were determined in Tg-RKIP1, Tg-RKIP2 and non-transgenic FVB mice by immunoblot detection (±s.d.; *n* = 8; ^***^p < 0.001 vs. FVB; Dunnett's test). **(B)** Immunohistological localization of RKIP on cardiac sections from an 8-months-old Tg-RKIP1 mouse and an age-matched non-transgenic FVB mouse (bar: 2 mm; nuclei were stained with hematoxylin, HE). Immunohistology is representative of four hearts/group. **(C,D)** Heart-to-body weight ratio **(C)**, and cardiac left ventricular ejection fraction **(D)** of 8-months-old Tg-RKIP1, Tg-RKIP2 and non-transgenic FVB mice [±s.d.; *n* = 6 **(C)**, and *n* = 5 **(D)**; ^***^p < 0.001 vs. FVB; Dunnett's test]. **(E)** Echocardiographic determination of left ventricular end-diastolic diameter (left), left ventricular end-systolic diameter (middle), and interventricular septal thickness (right) in 8-months-old Tg-RKIP1, Tg-RKIP2 and non-transgenic FVB control mice (±s.d.; *n* = 5; ^***^p < 0.001 vs. FVB; Dunnett's test). **(F)** Histological analysis of cardiac sections shows cardiac hypertrophy with dilatation in Tg-RKIP1 and Tg-RKIP2 hearts. **(G)**. Cardiomyocyte cross-sectional diameter **(G)**, systolic blood pressure **(H)**, and urinary albumin to creatinine ratio, ACR **(I)** in Tg-RKIP1, Tg-RKIP2, and non-transgenic FVB mice. **(J)** Increased body weight (left), lung weight (middle), and lung-to-body weight ratio (right) in Tg-RKIP1 and Tg-RKIP2 mice compared to non-transgenic FVB mice (±s.d.; *n* = 6; ^*^p < 0.05; ^**^p < 0.01; ^***^p < 0.001 vs. FVB; Dunnett's test). **(K,L)** 4 weeks of RKIP down-regulation by lentiviral transduction of an miRNA targeting RKIP by RNAi retards the development of cardiac dysfunction in 10-week-old (10 w) Tg-RKIP2 mice **(K)**. Panel **(L)** documents the down-regulation of cardiac RKIP (*PEBP1*) expression in miRKIP-transduced Tg-RKIP2 mice by qRT-PCR (±s.d.; *n* = 5; ^***^p < 0.001, ^**^p < 0.01, and ^*^p < 0.05 vs. FVB; Dunnett's test). See also Supplementary Figure 6.

Because cardiac effects induced by RKIP in mice could depend on the genetic background (35), we generated transgenic mice with a moderately increased cardiac RKIP protein level in the FVB background (Figure 7A). Two different transgenic lines were generated, which showed 2.7 ± 0.3-fold and 3.3 ± 0.4-fold increased cardiac RKIP protein levels over the non-transgenic FVB control (Figure 7A). Immunohistological analysis confirmed the increased cardiac RKIP protein content in an 8-months-old Tg-RKIP heart compared to the non-transgenic FVB control (Figure 7B).

Tg-RKIP mice with FVB background developed cardiac hypertrophy, which was detected by an increased heart-to-body weight ratio of 8-months-old mice (Figure 7C). Concomitantly, Tg-RKIP mice with FVB background showed symptoms of heart failure, which was documented by a severely decreased left ventricular ejection fraction of 30.4 ± 3.5% and 25.1 ± 4.7% in Tg-RKIP1 and Tg-RKIP2 mice (Figure 7D). Echocardiographic data confirmed that Tg-RKIP mice with FVB background had significant cardiac hypertrophy at an age of 8 months (Figure 7E). Cardiac enlargement was documented by significantly increased left ventricular end-diastolic and end-systolic diameters of Tg-RKIP compared to non-transgenic FVB mice (Figure 7E). As a control, interventricular septal thickness was not significantly different between study groups (Figure 7E).

Histological assessment of hearts from two different RKIP-transgenic mouse lines in FVB background complemented this phenotype and showed massive cardiac hypertrophy with dilatation (Figure 7F). Histomorphological analysis confirmed the phenotype of cardiomyocyte hypertrophy by a significantly increased cardiomyocyte cross-sectional diameter in Tg-RKIP mice compared to non-transgenic FVB mice (Figure 7G). Similarly to Tg-RKIP mice with B6 background (cf. Figure 4), aged Tg-RKIP mice with FVB background progressed to chronic congestive heart failure with a significantly elevated systolic blood pressure, proteinuria and an increased body weight with pulmonary congestion, which was documented by an increased lung weight and lung-to-body weight ratio (Figures 7H–J).

The RKIP-induced cardiac hypertrophy and cardiac dysfunction were (partially) prevented by down-regulation of RKIP with lentiviral transduction of an miRNA targeting the RKIP (*PEBP1*) by RNA interference (Figures 7K,L). These experiments confirm that the heart failure phenotype in Tg-RKIP mice was caused by transgenic expression of RKIP. The data also indicate that heart failure symptoms induced by an increased cardiac RKIP level can be counteracted by RKIP down-regulation. Because RKIP levels are increased in failing human hearts (21), this finding could be relevant to the human disease.

### RKIP Promotes Cardiac Lipid Overload in FVB Background

**Figure 8:**
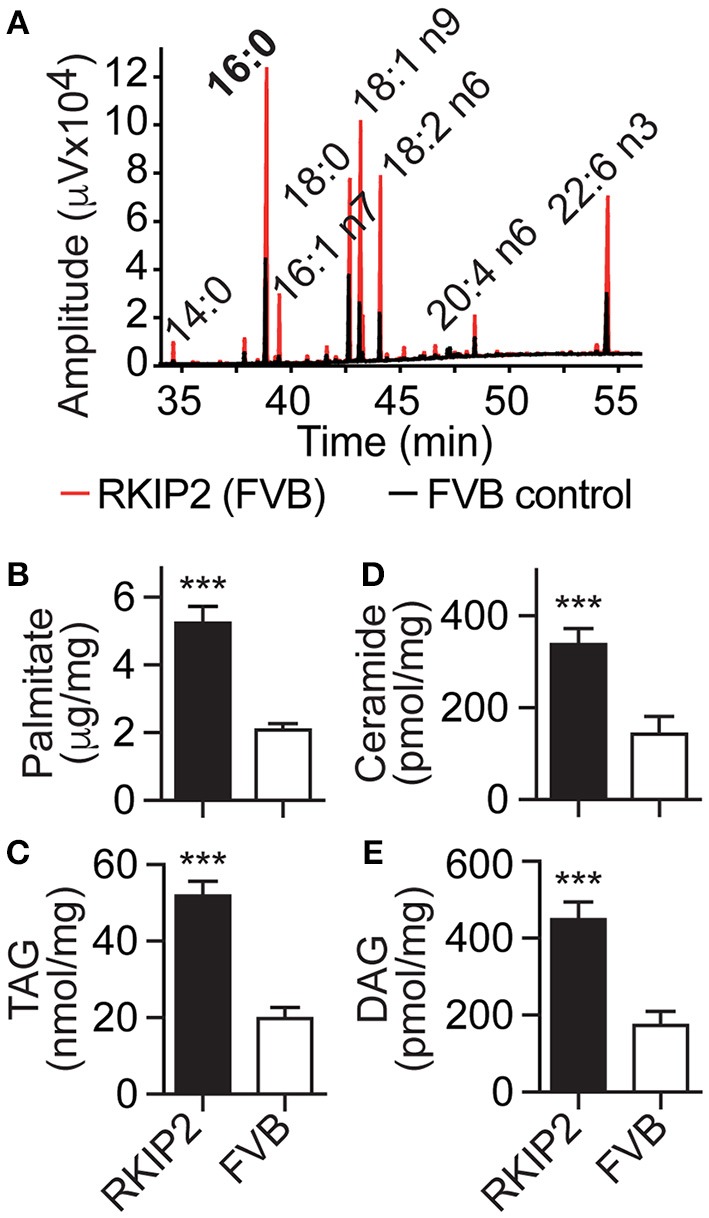
RKIP promotes cardiac lipid overload in FVB background. **(A–E)** GC analysis of cardiac lipids **(A)**, cardiac palmitate **(B)**, TAG **(C)**, ceramide **(D)**, and DAG **(E)** contents in 8-months-old Tg-RKIP2 mice with FVB background compared to age-matched non-transgenic FVB control mice (±s.d.; *n* = 6; ^***^p < 0.001). See also Supplementary Figure 7.

In addition to symptoms of chronic congestive heart failure, Tg-RKIP mice with FVB background also developed cardiac lipid overload (Figures 8A–E). Analysis of cardiac fatty acid composition by gas chromatography (GC) indicated that palmitate was the major lipid in Tg-RKIP hearts (Figures 8A,B). Other cardiotoxic lipid species such as ceramide and DAG were also significantly increased in Tg-RKIP hearts with FVB background (Figures 8D,E). Together these findings show that a moderately increased cardiac RKIP protein level is sufficient to promote symptoms of congestive heart failure, cardiac hypertrophy with dilatation and cardiotoxic lipid load in two different genetic backgrounds, i.e., B6 and FVB.

### RKIP and GRK2-K220R Act as GRK2 Inhibitors *in vivo*

**Figure 9:**
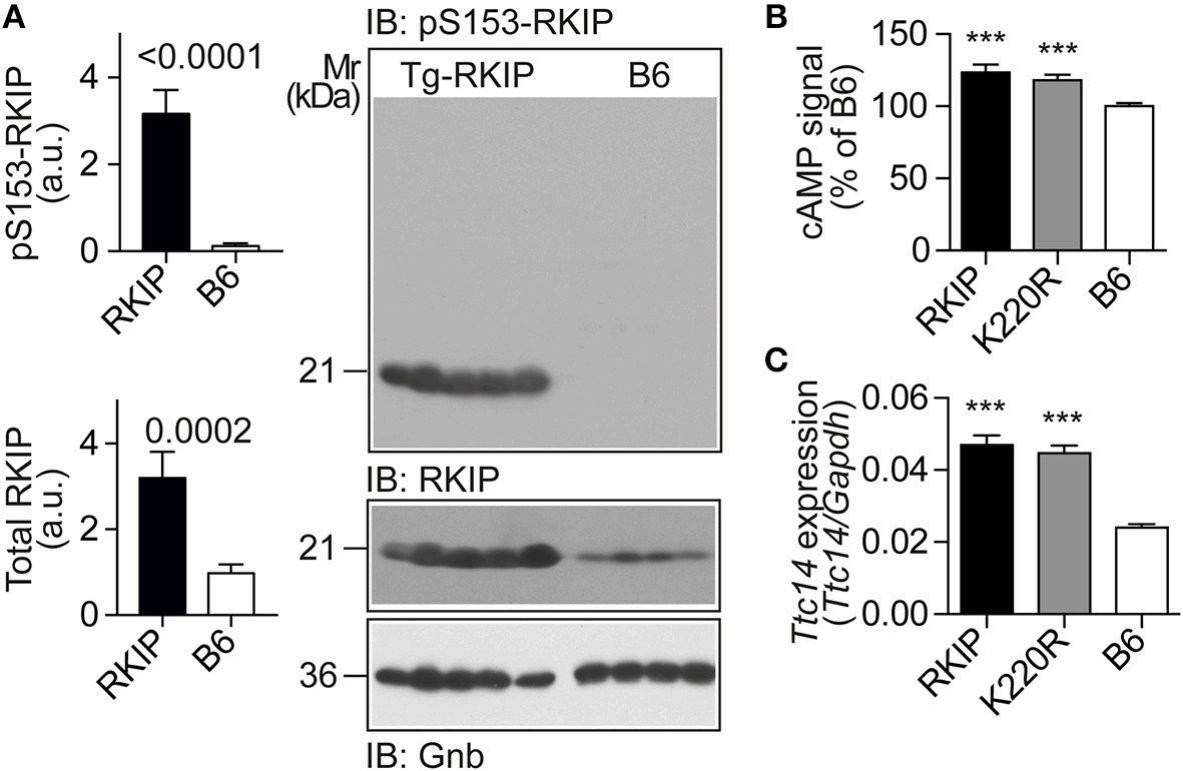
RKIP and GRK2-K220R act as GRK2 inhibitors *in vivo*. **(A)** Immunoblot detection of RKIP phosphorylation on serine-153 and total cardiac RKIP content in 8-months-old Tg-RKIP compared to non-transgenic B6 hearts (±s.d.; *n* = 5 Tg-RKIP; *n* = 4, B6). **(B,C)** Sensitization of the isoproterenol-stimulated cAMP response in neonatal cardiomyocytes isolated from Tg-RKIP and Tg-GRK2K220R mice compared to non-transgenic B6 cardiomyocytes **(B)**, and increased cardiac expression of the cAMP-inducible gene, *Ttc14*, in Tg-RKIP, and Tg-GRK2K220R hearts **(C)**. Data represent mean ±s.d. (*n* = 6, **B**; *n* = 3, **C**; ^***^p < 0.001 vs. B6; Dunnett's test). See also Supplementary Figure 8.

RKIP is a dual-specific inhibitor of GRK2 and the Raf-Erk1/2 axis (19). By PKC-mediated phosphorylation on serine-153, RKIP switches from Raf1 to GRK2 (19). Because the PKC-activating DAG was strongly increased in Tg-RKIP hearts, we investigated whether RKIP acted as GRK2 inhibitor *in vivo*, in Tg-RKIP mice with signs of heart failure. In agreement with an increased DAG load, immunoblot detection revealed a strongly increased content of serine-153-phosporylated RKIP in Tg-RKIP hearts compared to non-transgenic B6 controls (Figure 9A).

The serine-153 phosphorylation-dependent transformation of RKIP into a GRK2 inhibitor was accompanied by sensitization of cAMP signaling in isolated cardiomyocytes upon stimulation by the beta-adrenoceptor agonist, isoproterenol, (Figure 9B). In addition, sensitized cAMP signaling was also detected *in vivo* by a significant up-regulation of the cAMP-inducible gene, *Ttc14* [tetratricopeptide repeat protein 14; (36)], (Figure 9C). RKIP-mediated sensitization of cAMP signaling in Tg-RKIP cardiomyocytes and hearts was comparable to effects seen with the dominant-negative GRK2-K220R mutant in Tg-GRK2K220R cardiomyocytes and hearts (Figures 9B,C). Taken together, RKIP acts as GRK2 inhibitor *in vivo* and mediates sensitization of cAMP signaling similarly as the dominant-negative GRK2-K220R mutant.

### Concordant Gene Regulation in Tg-RKIP and Tg-GRK2K220R Hearts

**Figure 10:**
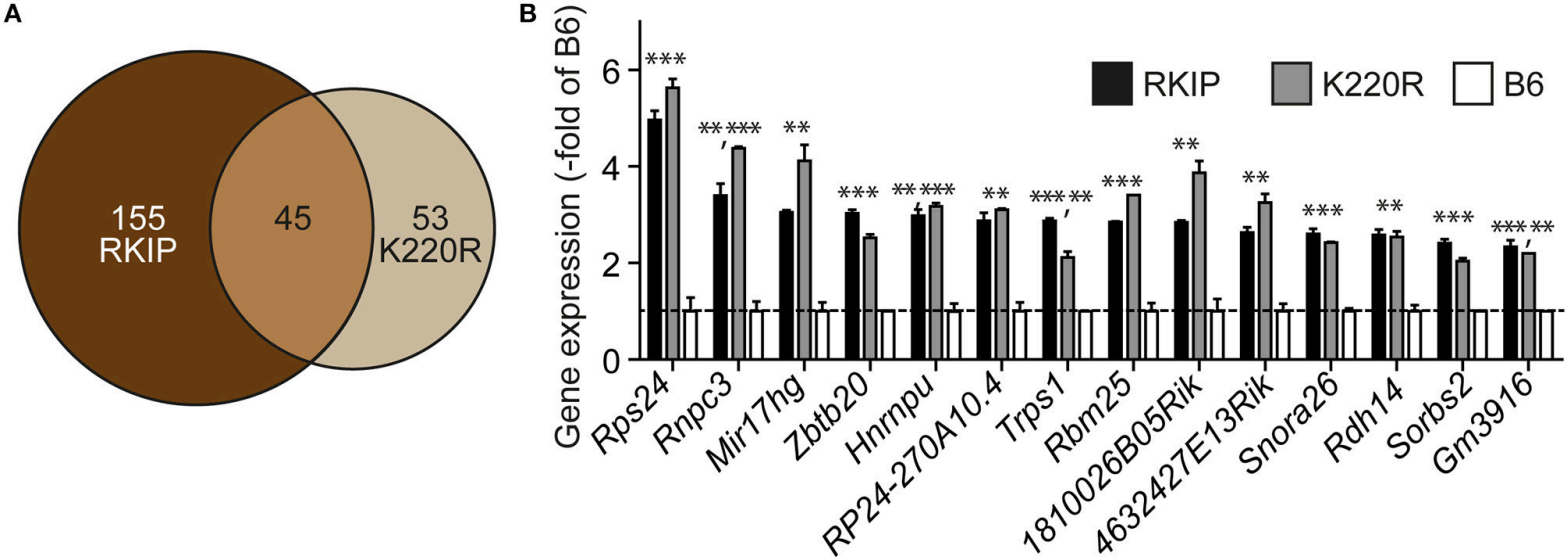
Concordant gene regulation in Tg-RKIP and Tg-GRK2K220R hearts. **(A,B)** Whole genome microarray gene expression profiling was performed with 8-months-old Tg-RKIP2 and Tg-GRK2-K220R hearts compared to non-transgenic B6 hearts. Probe sets with significantly different signal intensity (with call present and/or signal intensity >100) were identified with *p* < 0.01, and ≥2-fold difference compared to B6. The Venn diagram illustrates the number of concordantly regulated genes between Tg-RKIP and Tg-GRK2K220R hearts **(A)**. Representative probe sets **(B)** show comparable up-regulation of concordantly regulated genes in Tg-RKIP and Tg-GRK2K220R hearts (±s.d.; *n* = 2 gene chips/group with *n* = 3 hearts pooled for one gene chip; ^***^p < 0.001, ^**^p < 0.01 vs. B6; Dunnett's test).

We analyzed the impact of GRK2 inhibition by RKIP and GRK2-K220R on cardiac gene expression. Whole genome microarray gene expression profiling data showed concordant gene regulation in Tg-RKIP and Tg-GRK2K220R hearts (Figures 10A,B). Notably, 46% of all genes regulated by the dominant-negative GRK2-K220R mutant in Tg-GRK2K220R hearts were also concordantly regulated by RKIP in Tg-RKIP hearts (Figure 10A). These data further support the notion that moderate transgenic expression levels of RKIP and GRK2-K220R induce comparable GRK2 inhibition in transgenic mice.

### Dominant-Negative GRK2-K220R Retards Chronic Pressure Overload-Induced Cardiac Dysfunction

**Figure 11:**
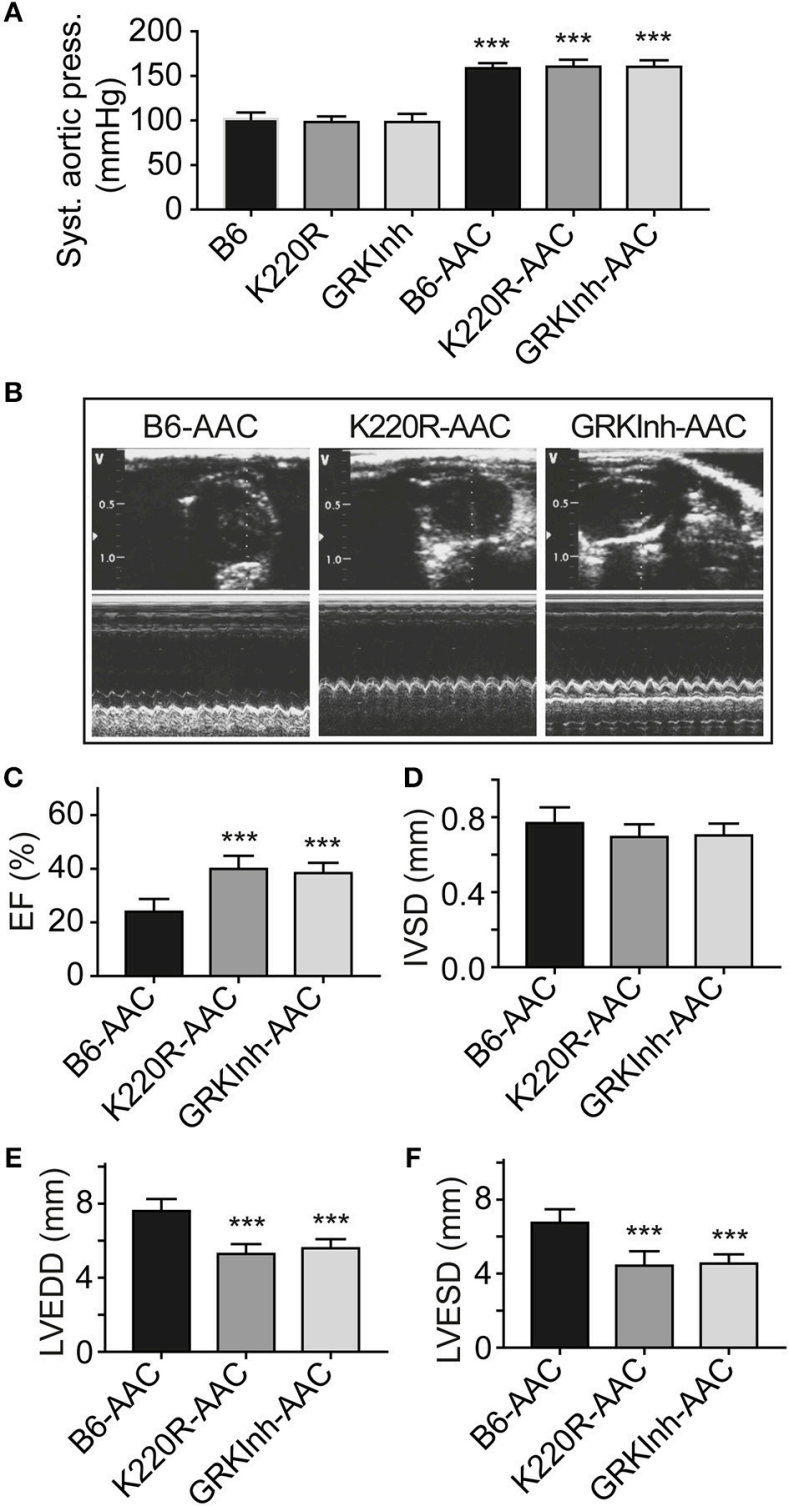
Dominant-negative GRK2-K220R retards chronic pressure overload-induced cardiac dysfunction. **(A)** Systolic aortic pressure of B6, Tg-K220R and Tg-GRKInh mice under basal conditions and at the end of the observation period after 2 months of AAC (±s.d.; *n* = 6; ^***^p < 0.001 vs. B6; Dunnett's test). **(B)** Representative echocardiographic images in 2D mode (upper) and M-mode (lower) of B6, Tg-GRK2K220R and Tg-GRKInh mice with 2 months of chronic pressure overload imposed by AAC. **(C–F)** Left ventricular ejection fraction **(C)**, interventricular septal thickness **(D)**, LVEDD **(E)** and LVESD **(F)** were determined by echocardiography of B6, Tg-GRK2K220R and Tg-GRKInh mice at the end of the observation period after 8 weeks of AAC (±s.d.; *n* = 6; ^***^p < 0.001; Dunnett's test).

We asked whether GRK2-K220R was cardio-protective in a model of heart failure induced by chronic pressure overload imposed by abdominal aortic constriction, AAC. As a control for cardioprotective GRK2 inhibition, we used Tg-GRKInh mice with myocardium-specific expression of a cardioprotective GRK2-inhibitory peptide derived from the first intracellular loop of the beta2-adrenoceptor, which is known to retard AAC-induced cardiac dysfunction (8, 12). Systolic aortic pressure of B6, Tg-GRK2K220R and Tg-GRKInh mice was comparable under basal conditions (Figure 11A). At the end of the observation period, AAC-induced chronic pressure overload was controlled and confirmed by a significantly increased systolic aortic pressure (>150 mmHg), which was not different between study groups (Figure 11A). The transgenic expression of dominant-negative GRK2-K220R in Tg-GRK2K220R mice significantly retarded the development of cardiac dysfunction induced by 8 weeks of AAC compared to non-transgenic B6 controls (Figures 11B,C). The cardioprotective effect of transgenic GRK2-K220R expression was comparable to GRK2 inhibition by transgenic expression of GRKInh (Figures 11B,C). Echocardiographic data further documented that inhibition of GRK2 by GRK2-K220R and GRKInh significantly inhibited the development of AAC-induced dilative cardiac hypertrophy, i.e., the left ventricular end-diastolic and end-systolic dimensions were significantly smaller in Tg-GRK2K220R and Tg-GRKInh mice compared to non-transgenic B6 mice whereas the interventricular septal thickness was not significantly different between study groups (Figures 11D–F). Together these findings present strong evidence that inhibition of GRK2 by dominant-negative GRK2-K220R exerts protection against cardiac dysfunction induced by chronic pressure overload.

### Inhibition of the AT1 Receptor Retards RKIP-Induced Symptoms of Heart Failure

**Figure 12:**
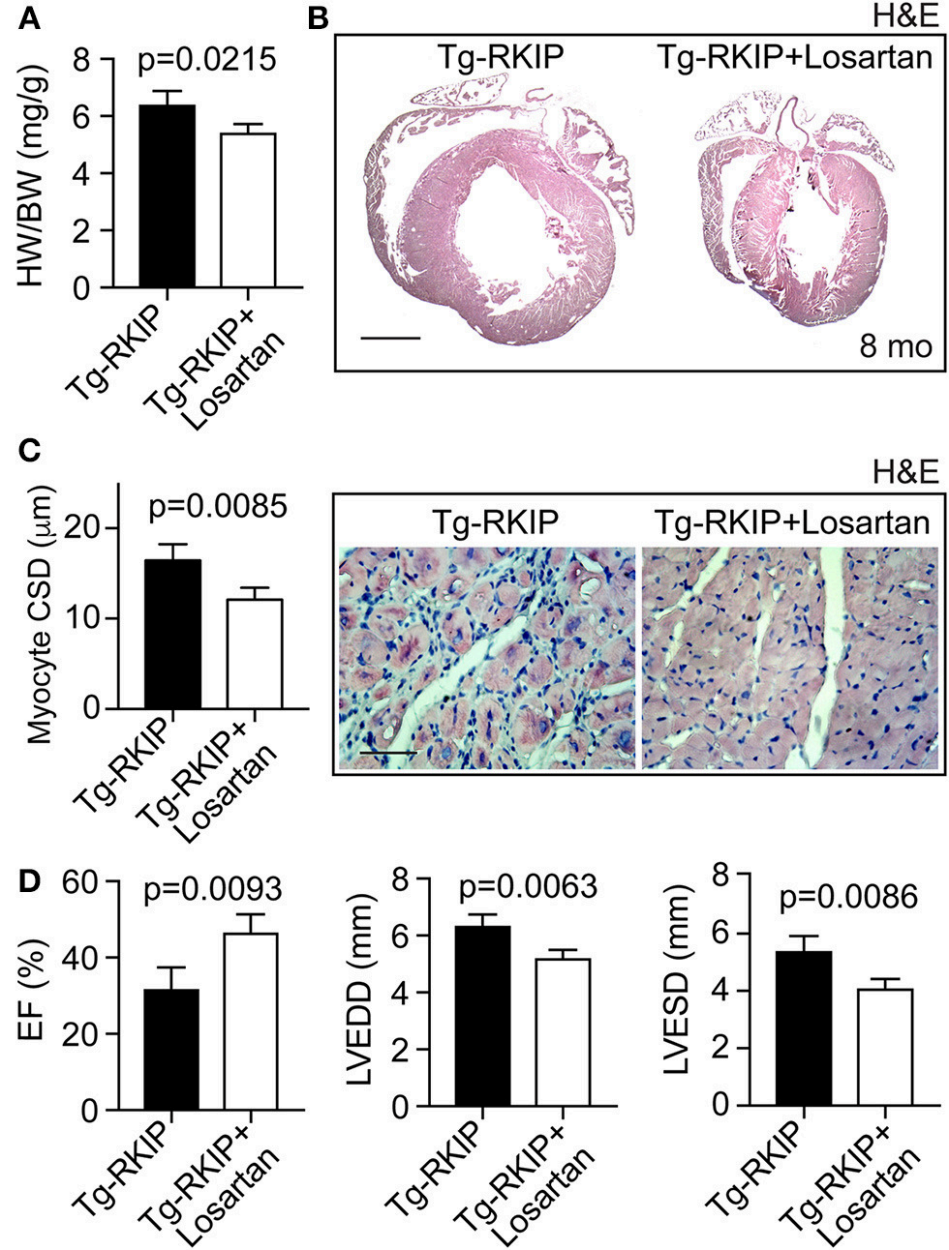
Inhibition of the AT1 receptor retards RKIP-induced symptoms of heart failure. **(A–C)** Treatment for 3 months with the AT1 antagonist, losartan, retards the development of RKIP-induced cardiac hypertrophy in 8-month-old Tg-RKIP mice with FVB background as evidenced by a decreased heart-to-body weight ratio **(A)**, histological analysis (**B**; bar: 2 mm), and cardiomyocyte cross-sectional diameter (**C**; bar 20 μm). Data represent mean ± s.d.; *n* = 4 **(A,C)**. Sections were stained with hematoxylin-eosin (H&E) and are representative of four mice/group **(B,C)**. **(D)** Left ventricular ejection fraction (left), left ventricular end-diastolic diameter (middle), and left ventricular end-systolic diameter (right) of Tg-RKIP mice without and with losartan treatment were determined by echocardiography (±s.d.; *n* = 4).

We finally investigated whether the AT1 receptor contributed to RKIP-induced symptoms of heart failure *in vivo*. To address this question, we treated Tg-RKIP mice for 3 months with the AT1-specific antagonist, losartan, at a dose of 5 mg/kg/day. The applied dose of losartan does not alter systolic blood pressure in mice (37). Treatment with losartan, significantly retarded the development of RKIP-induced cardiac hypertrophy as evidenced by a significantly decreased heart-to-body weight ratio, the histological assessment, and the histomorphological analysis of cardiomyocyte size (Figures 12A–C). Concomitantly, losartan treatment led to an improved cardiac function, which was documented by a significantly increased left ventricular ejection fraction (Figure 12D). Echocardiographic data further documented that inhibition of the pro-hypertrophic AT1 receptor by losartan treatment counteracted the phenotype of cardiac hypertrophy in Tg-RKIP mice (Figure 12D). Together these data provide strong evidence that sensitization of AT1 receptor signaling contributes to RKIP-induced cardiac hypertrophy and cardiac dysfunction.

## Discussion

In this study, we found that kinase-inactive GRK2K220R improves cardiac performance under basal conditions and exerts protection against chronic pressure overload-induced cardiac dysfunction. These findings with the GRK2-K220R mutant reflect the *in vivo* effects of a kinase-inhibited GRK2, which can be similarly achieved by a small molecule GRK2 inhibitor. In this respect our data with the kinase-inactive GRK2-K220R extend previous studies on the cardioprotective function of GRK2 inhibition, which is documented by different approaches of GRK2 inactivation, e.g., GRK2 deficiency (6), transgenic expression of the Gβγ-scavenging betaARKct (5, 9), the beta2-adrenoceptor-derived peptide, GRKInh (8, 12), and inhibition of GRK2 by an ATP-site-directed inhibitor (7).

Our study also shows that not all approaches of GRK2 inhibition are cardioprotective. In contrast to cardioprotective GRK2 inhibition with kinase-inactive GRK2-K220R, transgenic expression of the dual-specific GRK2 and Raf-Erk1/2 axis inhibitor, RKIP, promoted signs of heart failure, i.e., cardiac hypertrophy, cardiac dilatation and cardiotoxic lipid load. In addition, symptoms of chronic congestive heart failure were evident in Tg-RKIP mice as documented by pulmonary congestion with increased lung and body weight, kidney insufficiency with proteinuria, and elevated systolic pressure. RKIP-induced signs of heart failure were similarly observed in two different genetic backgrounds, i.e., B6 and FVB.

Signs of heart failure developed in RKIP-transgenic mice despite of significant GRK2 inhibition. Experimental data showed that GRK2 inhibition-induced sensitization of the cAMP signal was comparable between Tg-RKIP and Tg-GRK2K220R cardiomyocytes and hearts. Also, the phosphorylation of RKIP on serine-153 was significant in Tg-RKIP hearts, which is required for RKIP to act as GRK2 inhibitor. Nevertheless, the GRK2-inhibitory activity of RKIP was insufficient to protect against RKIP-induced heart failure.

In this study, several lines of evidence are presented, which show that sensitization of the AT1 receptor contributes to the observed RKIP-induced symptoms of heart failure. (I) RKIP led to increased AT1 receptor-stimulated inositol phosphate levels in cells. This AT1-sensitization was mediated by the RKIP-GRK2 interaction because the RKIP-S153V mutant, which cannot switch from Raf1 to GRK2, did not sensitize the AT1 receptor response. (II) RNAi-mediated down-regulation of RKIP demonstrated that endogenously expressed RKIP levels were sufficient to sensitize the AT1-stimulated signal. (III) Tg-RKIP mice with symptoms of heart failure showed down-regulation of the cardiac AT1 receptor content, which could be triggered by RKIP-mediated AT1 receptor sensitization because excessive AT1-mediated signaling down-regulates AT1 (31, 32). In this respect, Tg-RKIP mice resemble human patients with heart failure (29, 30). (IV) Tg-RKIP mice with myocardium-specific RKIP expression developed cardiac hypertrophy with dilatation, and cardiac dysfunction, and these symptoms of heart failure were retarded by treatment with the AT1-specific antagonist, losartan.

In addition to the heart failure-promoting effect of RKIP-triggered AT1 receptor sensitization, which was attributed to the RKIP-GRK2 interaction, symptoms of RKIP-induced heart failure could be aggravated by additional functions of RKIP. Notably, the severity of RKIP-induced symptoms of heart failure could be a consequence of the dual activity of RKIP as GRK2 and Raf1-Erk1/2 axis inhibitor. While AT1 receptor sensitization is largely mediated by the RKIP-GRK2 interaction, the development of cardiotoxic lipid load is attributed to Raf-Erk1/2 pathway inhibition, which leads to heart failure-promoting and adipogenic *Pparg* activation by inhibition of Pparg phosphorylation on serine-273 (8, 33). As a consequence, cardiotoxic lipid load occurs with accumulation of the PKC-activating DAG, which accounts for RKIP serine-153 phosphorylation and the RKIP-GRK2 interaction. The ensuing RKIP-GRK2 interaction not only restores the beta-adrenoceptor responsiveness but also triggers cardiotoxic AT1 receptor signaling. In addition, DAG-mediated activation of PKC is *per se* an independent contributor to heart failure (38).

Exaggerated cardiotoxic Gαq/11-stimulated calcium signaling seems to be a specific feature of RKIP because RKIP shields the amino-terminal domain of GRK2, which contains a cardioprotective and Gαq/11-inhibitory RGS domain (13, 14). In contrast to RKIP, established cardioprotective GRK2 inhibitors do not interfere with this RGS domain of GRK2. ATP-site-directed GRK2 inhibitors such as paroxetine, inhibit the kinase activity of GRK2 but leave kinase-independent functions of GRK2 intact, e.g., mediated by the Gαq/11-inhibitory RGS domain. Also, the betaARKct inhibits GRK2 by Gβγ subunit scavenging but retains the activity of the RGS domain (4).

The heart failure-promoting activity of RKIP in transgenic mouse models could also be relevant for the human disease because cardiac biopsy specimens from heart failure patients have an increased cardiac RKIP content (21). Notably, RKIP expression levels in several transgenic mouse models with two different genetic backgrounds, were comparable to the increased RKIP level found on human heart biopsy specimens of heart failure patients (21). Because down-regulation of an increased RKIP level in transgenic mice retarded the development of heart failure, RKIP inhibition could be considered as a potential target for development of a pharmacological treatment approach of heart failure. The recently elucidated beneficial and cell-protective side-effect profile of RKIP deficiency and/or RKIP inhibition further supports such a concept (39).

## Data Availability Statement

Whole genome expression data generated and analyzed for this study can be found in the NCBI GEO database (accession number GSE 120020).

## Author Contributions

SW and JA performed experiments. JA generated transgenic mice. UQ: conducted the study, designed experiments, and wrote the manuscript. All authors evaluated data, read and approved the final version of the manuscript.

### Conflict of Interest Statement

Contents of patent application WO/2018/130537 titled Cardioprotective compounds and their use, are related to contents of this manuscript. The handling editor and reviewer SK declared their involvement as co-editors in the Research Topic, and confirm the absence of any other collaboration.
