# Supplementary material for: Sensitization of the Angiotensin II AT1 Receptor Contributes to RKIP-Induced Symptoms of Heart Failure
Source: Front Med (Lausanne). 2019 Jan 9;5:359. doi: 10.3389/fmed.2018.00359 (PMC6333672; doi:10.3389/fmed.2018.00359)
Supplement: Supplementary file 1 [file Data_Sheet_1.PDF]

*Supplementary Material*

# Sensitization of the Angiotensin II AT1 Receptor Contributes to RKIP-Induced Symptoms of Heart Failure

**Stefan Wolf, Joshua Abd Alla, Ursula Quitterer***

*** Correspondence:** ursula.quitterer@pharma.ethz.ch

## 1 Supplementary Figures

Supplementary Figure 1

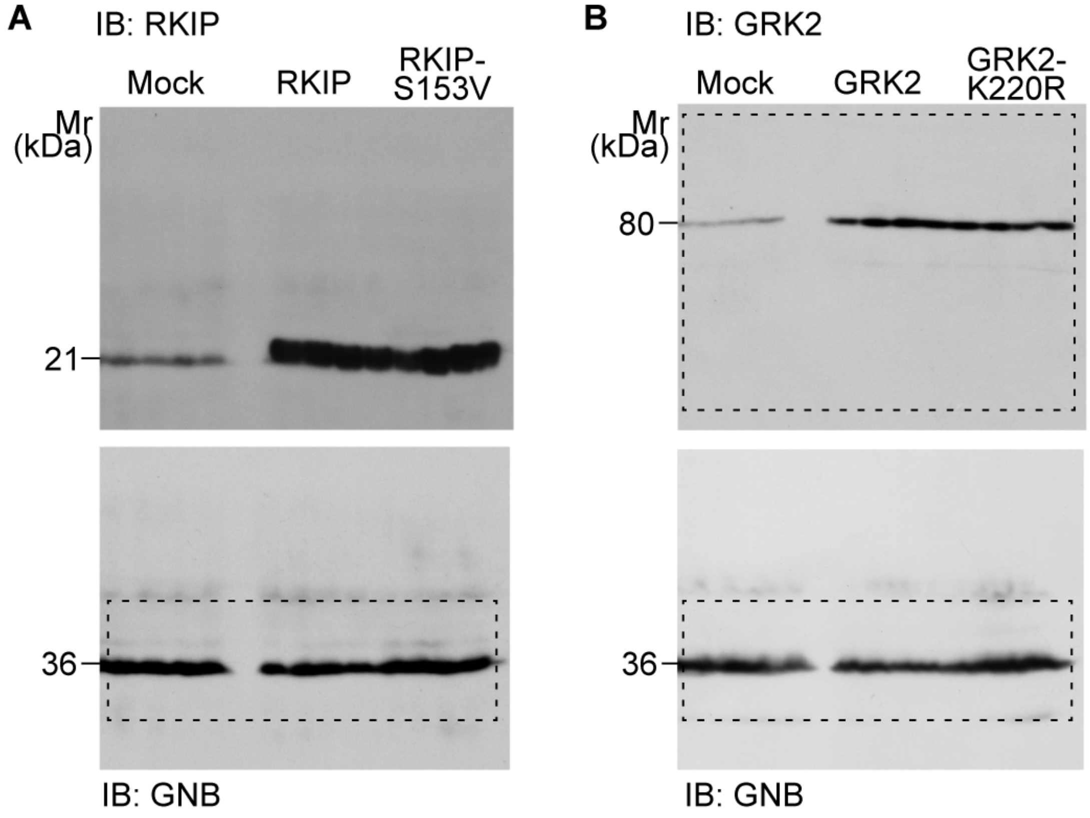


**Supplementary Figure 1.** (**A**) Immunoblots of Figure 1B. (**B**) Immunoblots of Figure 1C. (Related to Figure 1).

Supplementary Figure 2

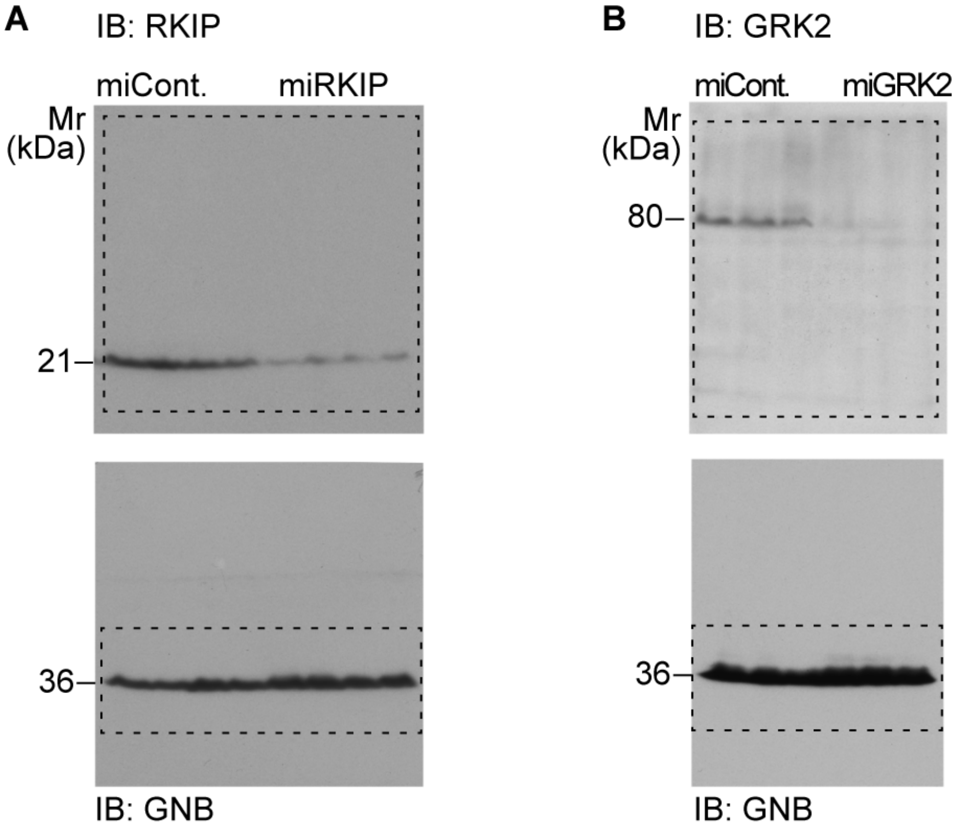


**Supplementary Figure 2.** (**A**) Immunoblots of Figure 2A. (**B**) Immunoblots of Figure 2B. (Related to Figure 2).

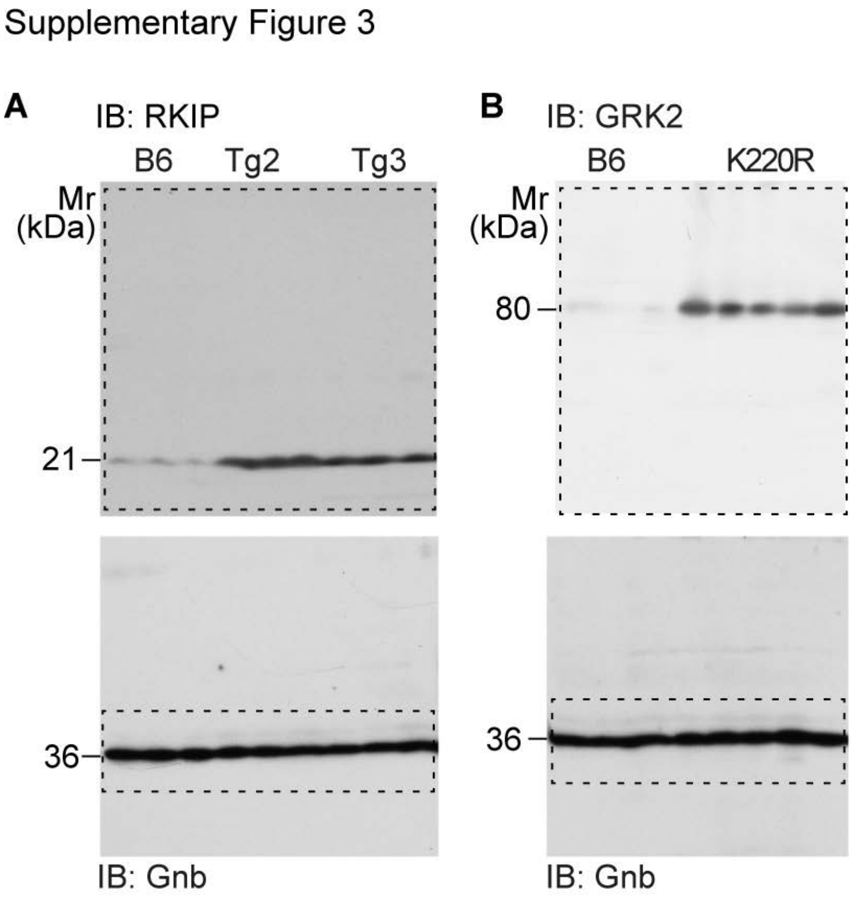


**Supplementary Figure 3.** (**A**) Immunoblots of Figure 3A. (**B**) Immunoblots of Figure 3B. (Related to Figure 3).

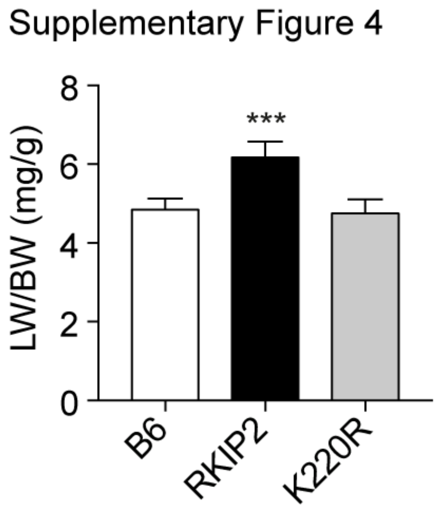


**Supplementary Figure 4.** Lung-to-body weight ratio of B6, Tg-RKIP2 and Tg-GRK2K220R mice (±s.d.; n=6; ***,$p<0.001$ vs. B6 and K220R; Tukey`s test).
(Related to Figure 4).

Supplementary Figure 5

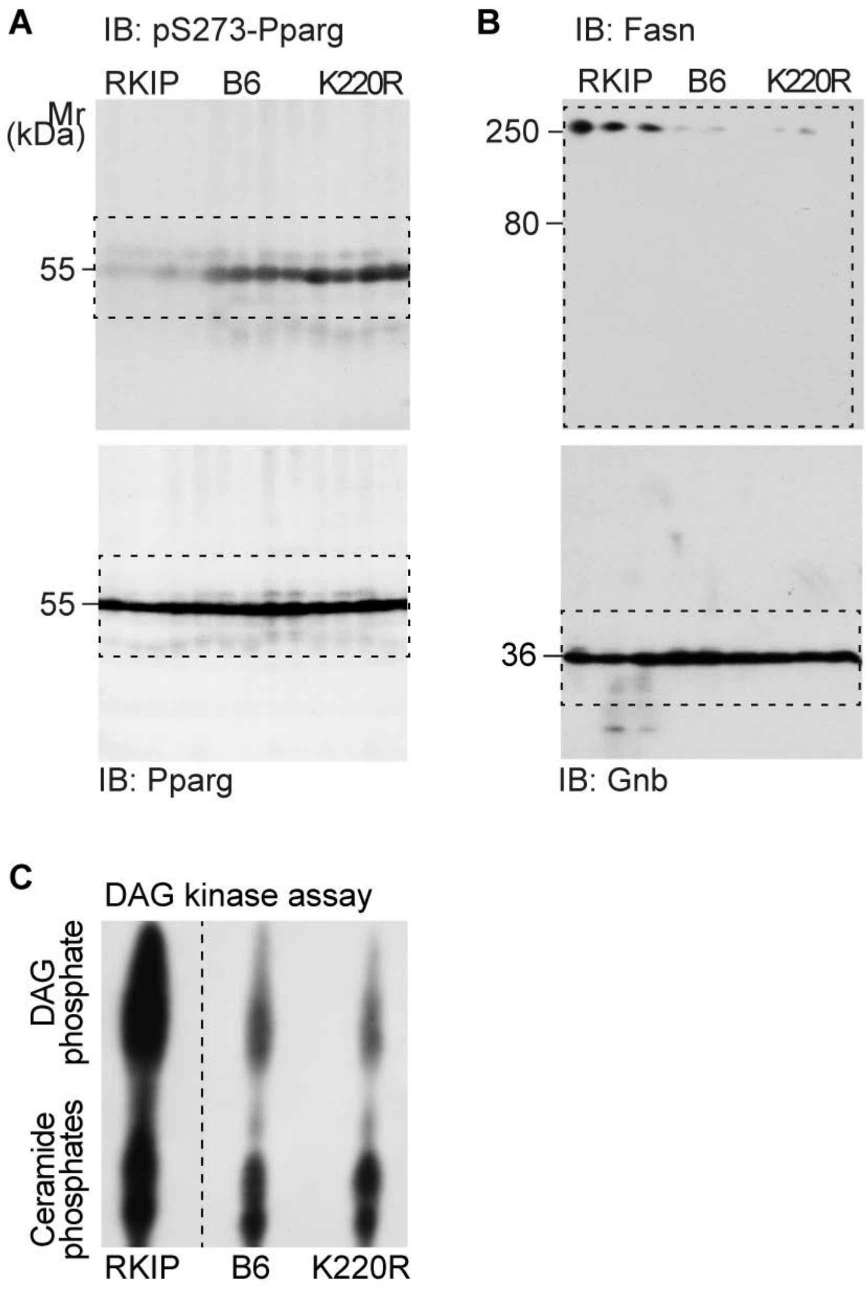


**Supplementary Figure 5.** (**A**) Immunoblots of Figure 6A. (**B**) Immunoblots of Figure 6B. (**C**) Autoradiograph of thin layer chromatography (TLC) detection of cardiac diacylglycerol (DAG) and ceramides upon phosphorylation by the DAG kinase shows increased cardiac DAG and ceramide contents in Tg-RKIP mice compared to B6 and Tg-GRK2-K220R mice. Quantitative data are shown in Figure 6G,H
(Related to Figure 6).

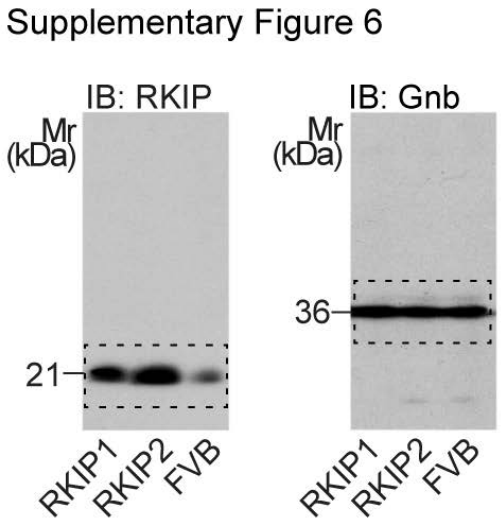


**Supplementary Figure 6.** Immunoblots of Figure 7A.
(Related to Figure 7).

Supplementary Figure 7

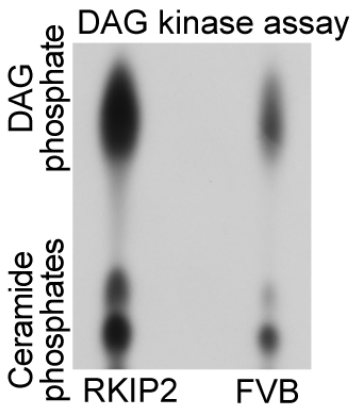


**Supplementary Figure 7.** TLC autoradiograph of DAG kinase assay of Figure 8D,E detects increased cardiac DAG and ceramides in Tg-RKIP2 mice with FVB background. (Related to Figure 8).

Supplementary Figure 8

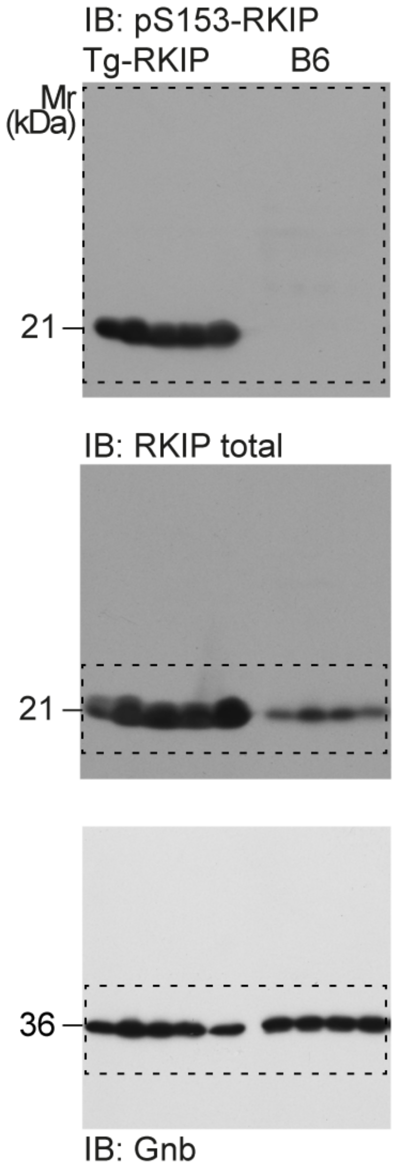


**Supplementary Figure 8.** Immunoblots of Figure 9A.
(Related to Figure 9).
